# Hydrogel-Integrated Millifluidic Systems: Advancing the Fabrication of Mucus-Producing Human Intestinal Models

**DOI:** 10.3390/cells13131080

**Published:** 2024-06-21

**Authors:** Ahed Almalla, Nadra Alzain, Laura Elomaa, Fiona Richter, Johanna Scholz, Marcus Lindner, Britta Siegmund, Marie Weinhart

**Affiliations:** 1 Institute of Chemistry and Biochemistry, Freie Universität Berlin, Takustr. 3, 14195 Berlin, Germanynadra.alzain@charite.de (N.A.); laura.elomaa@fu-berlin.de (L.E.); fior98@zedat.fu-berlin.de (F.R.); johascholz@zedat.fu-berlin.de (J.S.); m.lindner@fu-berlin.de (M.L.); 2 Department of Gastroenterology, Infectious Diseases and Rheumatology (Including Nutrition Medicine), Charité—Universitätsmedizin Berlin, Hindenburgdamm 30, 12203 Berlin, Germany; britta.siegmund@charite.de; 3 Institute of Physical Chemistry and Electrochemistry, Leibniz Universität Hannover, Callinstr. 3A, 30167 Hannover, Germany

**Keywords:** intestinal organoid, human terminal ileum, cell-based mucus model, physiological shear stress, CFD, dynamic cell culture, extracellular matrix, 3D printing, vat photopolymerization

## Abstract

The luminal surface of the intestinal epithelium is protected by a vital mucus layer, which is essential for lubrication, hydration, and fostering symbiotic bacterial relationships. Replicating and studying this complex mucus structure in vitro presents considerable challenges. To address this, we developed a hydrogel-integrated millifluidic tissue chamber capable of applying precise apical shear stress to intestinal models cultured on flat or 3D structured hydrogel scaffolds with adjustable stiffness. The chamber is designed to accommodate nine hydrogel scaffolds, 3D-printed as flat disks with a storage modulus matching the physiological range of intestinal tissue stiffness (~3.7 kPa) from bioactive decellularized and methacrylated small intestinal submucosa (dSIS-MA). Computational fluid dynamics simulations were conducted to confirm a laminar flow profile for both flat and 3D villi-comprising scaffolds in the physiologically relevant regime. The system was initially validated with HT29-MTX seeded hydrogel scaffolds, demonstrating accelerated differentiation, increased mucus production, and enhanced 3D organization under shear stress. These characteristic intestinal tissue features are essential for advanced in vitro models as they critically contribute to a functional barrier. Subsequently, the chamber was challenged with human intestinal stem cells (ISCs) from the terminal ileum. Our findings indicate that biomimicking hydrogel scaffolds, in combination with physiological shear stress, promote multi-lineage differentiation, as evidenced by a gene and protein expression analysis of basic markers and the 3D structural organization of ISCs in the absence of chemical differentiation triggers. The quantitative analysis of the alkaline phosphatase (ALP) activity and secreted mucus demonstrates the functional differentiation of the cells into enterocyte and goblet cell lineages. The millifluidic system, which has been developed and optimized for performance and cost efficiency, enables the creation and modulation of advanced intestinal models under biomimicking conditions, including tunable matrix stiffness and varying fluid shear stresses. Moreover, the readily accessible and scalable mucus-producing cellular tissue models permit comprehensive mucus analysis and the investigation of pathogen interactions and penetration, thereby offering the potential to advance our understanding of intestinal mucus in health and disease.

## 1. Introduction

The luminal surface of the intestinal epithelium is protected by a dynamic mucus layer that is essential for lubrication, water retention, and fostering symbiotic relationships with commensal bacteria [1]. The gel-like layer comprises water (98%) and mucins (2%), with minor constituents, including proteins, lipids, salts, DNA, and cellular debris. Mucins are high-molecular-weight glycoproteins with branched oligomeric carbohydrate side chains on an elongated protein backbone. Gel-forming mucins, including MUC2, MUC5B, and MUC5AC, assemble into a highly swollen hydrogel. MUC2 is the predominant gel-forming mucin in the gut, reaching several hundred micrometers on the human intestinal surface [2]. Membrane-associated mucins, including MUC1, MUC3A, MUC3B, and MUC4, serve to tether the gel to the epithelial cell surface [3].

Despite critically contributing to the first line of defense in the intestinal lumen, the precise functions of the mucus layer in health and disease are not yet fully understood [4]. The challenges of accessing [5,6], processing, and preserving intestinal mucus [7], along with the variability between and within organs [8], impede systematic investigation and controlled modulation. In vitro intestinal models offer a promising avenue for addressing these challenges, provided they accurately replicate the composition and properties of human intestinal mucus, particularly health- and disease-specific features.

Current state-of-the-art models utilize human intestinal stem and progenitor cells (ISCs) isolated from crypts, capable of differentiating into a variety of intestinal epithelial cell types, including mucus-producing goblet cells. In contrast to intestinal cell lines, such as HT29-MTX, which exhibit a mixed absorptive goblet cell phenotype after differentiation and primarily secrete the gastric-specific mucin MUC5AC [9], ISC-derived goblet cells mainly secrete the intestine-specific mucin MUC2 [10]. When cultured in Matrigel^TM^, ISCs assemble into proliferative organoid structures that resemble the intestinal lumen in their interior. These structures serve as models and tools for basic research and personalized medicine [11,12,13]. Nevertheless, 3D-cultured organoids exhibit limited mucus accessibility, as the secreted mucus lining the lumen appears entrapped within the organoid, impeding further manipulation [14,15]. To address this, apical-out repolarized intestinal organoids, which currently do not qualify for maintenance culture [16], and more practical 2D monolayers obtained from reseeding organoid-derived ISC fragments or single cells have been developed [17]. As substrates for two-dimensional (2D) models, various synthetic materials are employed, including non-porous tissue culture-treated polystyrene (TCPS) [18] and porous membranes derived from polycarbonate, polyethylene terephthalate (PET) [19], or polydimethylsiloxane (PDMS) [14]. The substrates are typically coated with a biomimetic thin layer of extracellular matrix (ECM)-derived proteins, such as growth factor-reduced Matrigel^TM^, collagen, or laminin, to support ISC adhesion and growth. These substrates offer essential mechanical support, yet they do not fully recapitulate the biochemical and biophysical features of the compartmentalized intestinal microenvironments. Consequently, human ISCs seeded on 2D substrates frequently form fractured monolayers with a limited lifespan. These monolayers lack the critical self-sustainment necessary for long-term culture following a transition from an expansion to a differentiation medium [19]. The loss of stemness during the formation of a 2D monolayer or the differentiation of ISCs in vitro can be attributed to unphysiological substrate stiffness and inappropriate regulatory signaling [20,21]. Soft, bulk hydrogel substrates (Young’s modulus: <1 kPA) made from natural ECM components, such as collagen or Matrigel^TM^ [22], more closely replicate the composition and stiffness of the basal membrane of the intestinal stem cell niche, thereby supporting the development and proliferation of ISC-based self-sustained monolayers [21,23]. Despite their benefits, such physically crosslinked hydrogels hardly support long-term cultures due to their rapid enzymatic degradation and mechanical instability. To provide enhanced structural and mechanical support, such biopolymer-based gels require additional chemical/covalent crosslinking, particularly when 3D structuring of the bulk hydrogel scaffolds is desired [24]. Consequently, many laboratories currently rely on organoid-derived ISC monolayers cultured on Matrigel^TM^-coated synthetic membranes due to the convenient and straightforward preparation process.

Chemical differentiation protocols are commonly used to induce ISC differentiation [11,12,19]. Recently, alternative mechanical stimulation through peristalsis or fluid shear stress has emerged as an effective and more physiological alternative to differentiating ISCs [14,25] and cell lines into multiple intestinal cell types [26,27], often accompanied by 3D reorganization of the monolayer. Piezo1, a mechanical sensor expressed on epithelial cells [28], plays a pivotal role in this process by regulating stem cell proliferation and differentiation [29], tight junction protein expression [30], and MUC2 production and secretion of goblet cells [31].

In light of the current opportunities in biofabrication, we propose the development of advanced mucus-producing ISC-based models utilizing 3D-printed biomimetic hydrogel scaffolds with adjustable stiffness and the application of shear stress for the purpose of tissue maturation and mucus modulation. In order to facilitate dynamic culture, a tissue chamber was designed that hosts multiple cell-seeded hydrogels for exposure to adjustable apical shear stress. The chamber’s performance was validated with HT29-MTX cells, and its efficacy in the solely shear stress-triggered multi-lineage differentiation of human ISCs from the terminal ileum was demonstrated, thereby highlighting the beneficial mucus-related effects achievable in a dynamic culture environment.

## 2. Materials and Methods

### 2.1. Materials

All chemicals, buffers, and reagents utilized in this investigation were acquired and prepared as previously published [26,32,33]. The Supplementary Materials (SM) section provides a comprehensive listing of these materials.

### 2.2. Decellularized and Methacrylated Small Intestinal Submucosa (dSIS-MA): Preparation, Printing, and Characterization

The preparation of dSIS-MA as a biomaterial resin for vat photopolymerization involved the decellularization, solubilization, and methacrylation of small intestinal submucosa (SIS) for subsequent 3D printing into hydrogels 6 mm in diameter (*d*) and 1 mm in height (*h*). The diameter of the scaffolds was chosen to align with the dimensions of standard 96-well plates (*d* = 6.4 mm per well) for static culture controls and the cavities of the tissue chamber for dynamic culture following cell seeding. Their precise geometry enabled them to occupy the surface area of a well or tissue chamber’s cavity seamlessly while still allowing for easy sample handling within a small margin of space. The preparative procedures, along with a comprehensive characterization of the resulting resin and the printed dSIS-MA-based hydrogels, were performed in accordance with our established protocols and are briefly outlined in the SM [32,33]. The characterization of materials was achieved through a number of different methods, including nuclear magnetic resonance spectroscopy (^1^H NMR), circular dichroism spectroscopy (CD), rheology, atomic force microscopy (AFM), scanning electron microscopy (SEM), and chromogenic assays.

### 2.3. Flow Chamber Design, Fabrication, and Computational Fluid Dynamics (CFD)

**Figure 1:**
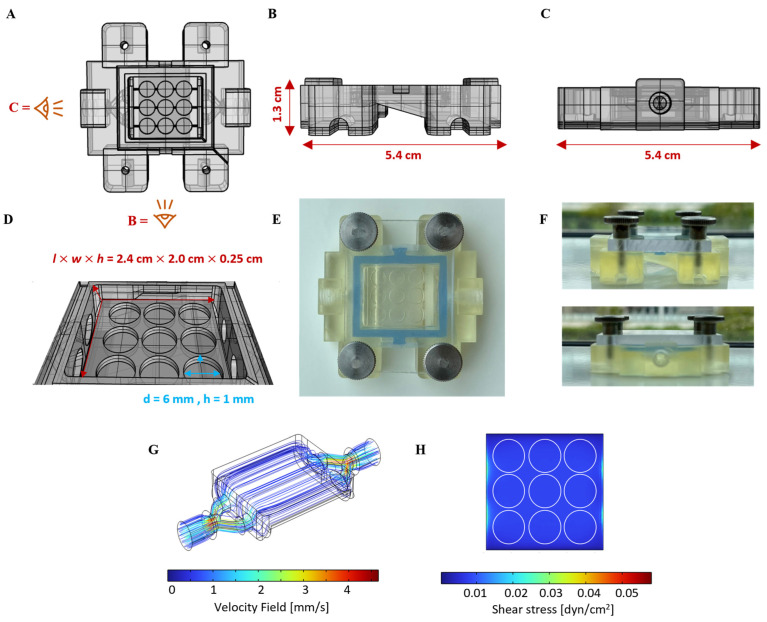
Illustration of flow chamber design to hold multiple surface-cellularized, dSIS-MA hydrogel scaffolds and apply defined, physiological surface shear stress under dynamic cell culture conditions. (**A**) Computer-aided design (CAD) model of the flow chamber body configured to accommodate nine scaffolds (d = 6 mm and h = 1 mm). (**B**,**C**) Side views of the chamber body with its dimensions. (**D**) Flow channel dimensions in the CAD model. (**E**) Photographic image of the fully assembled chamber body, including transparent lid, blue silicon sealing, and four M3 screws. (**F**) Side views of the assembled chamber in (**E**). (**G**,**H**) CFD simulation of surface shear stress within the flow channel, with color gradients representing uniform low flow velocity at a defined medium flow rate of 1.42 mL/min and consistent laminar shear stress distribution of around 0.01 dyne/cm^2^, indicating a homogenous environment for low fluid shear stress-assisted cell culture.

The body of the dynamic tissue culture chambers was designed using the 3D computer-aided design (CAD) software, Rhinoceros (Version 5.0, Robert McNeel & Associates, Seattle, WA, USA), and fabricated with a vat digital light projection (DLP) 3D printer (Anycubic Photon D2 from Anycubic Technology Co., Shenzhen, China). The STL files of the CAD models are provided as Supplementary Data. We utilized a biocompatible, autoclavable resin (BioMed Amber, Formlabs, Somerville, MA, USA) for printing, with an 8 s curing time for each 100 µm layer. Post-printing, the chamber bodies were thoroughly washed in isopropanol to remove uncross-linked resin and then post-cured under a 405 nm blue light lamp for 2.5 min on each side (Figure 1A). The printed chamber body dimensions were set at 5.4 cm × 5.4 cm × 1.3 cm (*l* × *w* × *h*) (Figure 1A–C). The chamber was designed with a centered, broad flow channel (*l* × *w* = 2.4 cm × 2.0 cm) 2.5 mm in height (Figure 1D). Located just below the flow channel were nine disk-like cavities (*d* = 6 mm, *h* = 1 mm) designed to accommodate the intestinal tissue models, which are intended to be exposed to fluid flow shear stress (Figure 1D). The flow channel is formed between the chamber body and a lid when closing the tissue chamber. A transparent polycarbonate (Makroclear^®^ from Arla Plast, Motala, Sweden) lid (*l* × *w* = 3.5 cm × 5.4 cm) with a height of 4 mm was precisely manufactured via computer numerical control (CNC) machining featuring four threads (M3, *d* = 3 mm), allowing it to be securely fastened to the chamber body with stainless-steel knurled thumb or socket head cap screws (M3, 1.2 mm, DIN 646, ERIKS, Halle (Westfalen), Germany) (Figure 1E,F). Detailed dimensions of the lid can be found in the SM. Tight sealing between the lid and chamber was accomplished through a silicone gasket custom-molded from a blue medical-grade two-component silicone kit (REPLISIL 22 N, SILCONIC^®^, Lonsee, Germany) (Figure 1E,F). After fabrication, additional hand-screwed threads (M5 screws) were created on the two sides of the chamber body where the fluid flow enters and exits the chamber to allow for the mounting of adapters (#40101, threaded connector, female luer lock, Qosina, Ronkonkoma, NY, USA) for tubing. The established flow chamber assembly was reusable and maintained structural integrity for up to 10 autoclaving cycles.

CFD analysis to simulate flow conditions, encompassing velocity fields and shear stress (Figure 1G,H), was conducted using COMSOL Multiphysics (v5.5, CFD module, COMSOL, Stockholm, Sweden). The laminar flow physics was selected, including gravity, and water was chosen as the fluid material. The inlet flow properties were set to 1.42 mL·min^−1^, with zero static pressure defined as the outlet condition. The default mesh setting was a “physics-controlled” mesh with a “normal” element size, and the mesh quality was judged based on COMSOL’s minimum element quality.

Wall shear stress (*τ*) within the chamber’s flow channel was estimated using Equation (1), as follows:

*τ* = 6*µQh*^−2^*w*^−1^ (1)

where *µ* denotes dynamic viscosity (*µ* = 0.93 mPa s [34]), *Q* is the volume flow rate [cm^3^s^−1^], and *w* and *h* denote the width [mm] and height [mm] of the fluidic channel, respectively.

### 2.4. Static Cell Culture

#### 2.4.1. Culture and Seeding of HT29-MTX Cells

Human HT29-MTX cells (European Collection of Authenticated Cell Cultures via Sigma Aldrich, Darmstadt, Germany) were cultured in high glucose Dulbecco’s Modified Eagle Medium (DMEM) enriched with 10% fetal bovine serum (FBS), 1% non-essential amino acid solution (MEM-NEAA), and 1% penicillin/streptomycin (p/s) antibiotic mixture with medium change every 2–3 days. Cultivation was carried out in an incubator at 37 °C with 5% CO_2_ and 95% humidity. To prepare 3D-printed dSIS-MA hydrogels (*d* = 6 mm, *h* = 1 mm) for cell seeding, they were disinfected with 70% ethanol for an hour and then washed three times in sterile phosphate buffered saline without calcium and magnesium ions (PBS −/−) with 1× p/s, with each wash lasting an hour. The disinfected hydrogels were placed in a 96-well plate (Sarstedt AG & Co., Nümbrecht, Germany) and stored in sterile PBS (−/−) at 4 °C until cell seeding. Before seeding, the PBS was carefully removed, and the hydrogels were allowed to equilibrate to room temperature for 10 min to prevent floating when the cell suspension was added. Next, HT29-MTX cells (passage numbers 10–13) were seeded at a density of 6 × 10^4^ cells per hydrogel (1.8 × 10^5^ cells/cm^2^) by carefully adding 100 μL of cell medium suspension dropwise directly to the center of each hydrogel to promote even cell distribution across the hydrogel surface. This setup was maintained for 5 days with medium changes every 2–3 days under static conditions to achieve a confluency between 80 and 100%. After reaching confluency, cellularized dSIS-MA hydrogels were transferred to the cavities of the flow chambers with the monolayers facing upward, marking the beginning of the experimental period (Day 0).

#### 2.4.2. Crypt Isolation and Organoid Formation and Expansion

The basal medium for crypt isolation and Matrigel™ dilution is composed of Gibco™ advanced Dulbecco’s Modified Eagle Medium (DMEM/12) supplemented with 2 mM Corning™ cellgro™ L-glutamine (Corning Incorporated, Kaiserslautern, Germany), 1× p/s, and 10 mM Gibco™ HEPES (Thermo Fisher Scientific, Darmstadt, Germany). Human primary epithelial cells were obtained from a biopsy of the terminal ileum of an adult healthy donor (sex: male; age: 20; approved by the ethical committee of the Charité—Universitätsmedizin Berlin). L-WRN cells (CRL-3276^TM^, ATCC^®^) were cultured to generate a conditioned medium containing the growth factors Wnt3a (W), R-spondin-3 (R), and Noggin (N) essential for the culture of human intestinal organoids, following the protocol established by Miyoshi and Stappenbeck [35]. The pooled WRN-conditioned medium was mixed in a 1:1 *v*/*v* ratio with PCM medium (Gibco™ advanced DMEM/F12 supplemented with Corning™ cellgro™ L-glutamine, 1× p/s, and 20% FCS). For undifferentiated organoid formation/expansion and ISC culture, the 1:1 medium mixture was further supplemented with recombinant human epidermal growth factor (EGF, 50 ng/mL), nicotinamide (10 mM), A83-01 (0.5 µM), SB202190 (10 µM), and human gastrin I (10 nM) to the specified final concentrations and is henceforth referred to as expansion media. Crypt isolation from biopsies and subsequent formation and expansion of intestinal organoids were performed as described earlier [33] and stated in the SM for reference. Passage numbers between 7 and 12 were used for the experiments.

#### 2.4.3. Culture of Organoid-Derived ISCs on dSIS-MA Hydrogels

For monolayer culture of organoid-derived ISCs, undifferentiated organoids from the human terminal ileum were isolated from Matrigel^TM^ and enzymatically dissociated with TrypLE™ supplemented with rock inhibitor (RI) Y-27632 (10 µM; #M1817, AbMole Bioscience, Houston, TX, USA) for 5 min at 37 °C, as detailed in the SM for passaging, and subsequently subjected to mechanical disruption by expelling the solution through a blunt 18G needle (8–12 times). The washed and isolated (400× *g*; 5 min; 4 °C) small ISC clusters containing around 10 cells per cluster and singularized cells were resuspended in expansion media supplemented with Y-27632 (10 µM) and shortly kept on ice until seeding. PBS was removed from disinfected dSIS-MA hydrogels (cf Section 2.4.1), and the hydrogels were equilibrated using a 20 min incubation in expansion media (50 μL) at 37 °C. After removing the cell media, hydrogels were left at 37 °C for 10 min before ISC seeding (1 × 10^5^ cells per hydrogel or ~3 × 10^5^ cells/cm^2^) to prevent scaffold floating.

Control experiments on conventional porous polyethylene terephthalate (PET) insert membranes (Falcon^®^ #353095, *d* = 6.5 mm, 0.4 µm pore size, Corning Inc., Kaiserslautern, Germany) were prepared in 24-well plates. The membranes were coated with Matrigel™ (100 μL, diluted 1:100 in basal medium, cf Section 2.4.2), which was solidified at 37 °C for 1 h prior to cell seeding. Alternatively, the dilute Matrigel™ solution was left on the membrane overnight at 4 °C and incubated at 37 °C for 20 min before seeding. After removing the residual solution, ISCs were seeded at the same density as described for the dSIS-MA hydrogel scaffolds.

For both substrates, the expansion media was replaced every 2–3 days, with the RI supplement excluded from the media after the initial seeding. Monolayers were cultivated for 2–3 days until reaching a confluency of 80–100%, after which they were subjected to fluid flow shear stress or maintained statically, indicated as “Day 0” in the experimental timeline.

### 2.5. Dynamic Cell Culture

**Figure 2:**
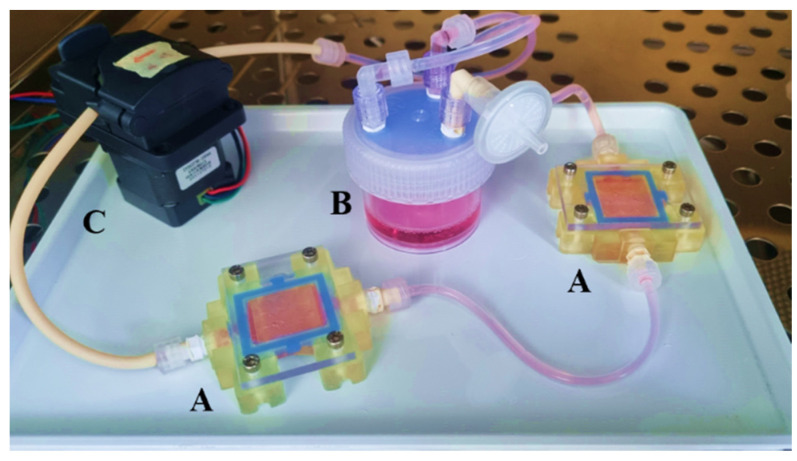
Photograph of the fully assembled dynamic cell culture circuit, including (A) two 3D-printed tissue chambers, (B) a medium reservoir filled with cell culture media and equipped with a sterile air filter for gas exchange, and (C) a peristaltic pump.

The dynamic cell culture setup includes a peristaltic pump (9QS, Boxer GmbH, Ottobeuren, Germany) connected to a medium reservoir containing inlet, outlet, and gas exchange adapters (15 mL, #10454631, PPCO, Nalgene™, Thermo Fisher Scientific, Langenselbold, Germany) and two customized 3D-printed chambers (Figure 2). For detailed fabrication of the media reservoir, see the SM. The system is connected via transparent silicone tubing (TYGON^®^ 3350, Platinum-Cured Silicone, France) with an inner diameter (ID) and wall thickness (WT) of 1.6 mm and 0.8, respectively, along with pump tubing made of a polypropylene-based thermoplastic elastomer (#P60, Innovaprene^®^, Suzhou Innovapure Co., Leyu Zhen, Suzhou, China) with ID = 2 mm and WT = 1 mm, linking the three components of the circuit. The pump is controlled via an Arduino-based open-source hardware platform. Step-by-step instructions for assembling the Arduino-controlled pump system and its operation, including all supplier specifications, can be found in the SM. The free Arduino IDE software (v 1.8.9) was employed to program the microcontroller via a customized script (see SM and additional INO file as Supplementary Data). For sterilization, the culture circuit (excluding the pump) is packaged in sterilization Steriking^®^ pouches (Wipak, Walsrode, Germany), sealed, and autoclaved at 120 °C. Medium exchange (12 mL) is performed every seven days with the dual-chamber setup (18 hydrogels in total, equivalent to a cell-seeded surface area of 5.09 cm^2^). The peristaltic pumps are calibrated to maintain a constant perfusion rate of 1.42 mL/min at 12 RPM through tubes while operating inside a cell culture incubator at 37 °C, 5% CO_2_, and 95% humidity. This setup yields a physiological surface shear stress of 0.01 dyne/cm^2^ on the cellularized hydrogels, referred to as “dynamic culture conditions” in the following sections.

### 2.6. Immunofluorescence Staining

Sample preparation and conditions for immunofluorescence staining can be found in the SM.

### 2.7. Alkaline Phosphatase (ALP) Activity Assay

Alkaline phosphatase (ALP) activity was measured at days 0, 3, 5, and 7 of a static and dynamic culture using an alkaline phosphatase assay fluorometric kit (#ab83371, Abcam, Regensburg, Germany), according to the manufacturer’s protocol. In static culture, the supernatants of twelve cellularized hydrogel or membrane samples were collected and combined for each group just before the scheduled media change on the specified days. In the dynamic culture, the cell medium (1 mL per 18 hydrogel samples) was collected from the media reservoir. All samples were snap-frozen in liquid nitrogen and stored at −80 °C until further analysis.

For the assay, samples (1 mL) were thawed on ice and transferred to a black 96-well plate (130 µL, Corning^TM^, Merck KGaA, Darmstadt, Germany). The results were normalized to account for the number of samples in each condition (12 in static vs. 18 in dynamic), the actual seeding area per sample (28.27 mm^2^ for hydrogels and 33.18 mm^2^ for membranes), and the initial medium volume (1.2 mL for static and 12 mL for dynamic conditions). The assay was performed with supernatants from three individual runs and reported as mU/mL ALP activity ± standard deviation (SD) following normalization and referred to an overall culture area of 3.4 cm^2^ per ISC monolayer, assuming similar cell density and size independent of the culture conditions and the substrate.

### 2.8. Mucus Quantification

#### 2.8.1. Sample Preparation for Quantification of Mucins in Supernatants

Cell medium (1 mL) was collected collectively per sample group and stored as described in Section 2.7 from supernatants under static conditions and from the medium reservoir under dynamic conditions on days 0, 3, 5, and 7 of culture. Grouped samples were collected from three individual runs and normalized for each run on the respective sample size, seeding area, and initial medium volume as described in Section 2.7 and reported as mg mucin per mL supernatant ± SD produced by a monolayer of ISCs on an overall culture area of 3.4 cm^2^.

#### 2.8.2. Sample Preparation for Quantification of Cell-Bound Mucins

First, culture media was removed, and the cells were washed with PBS (−/−) for both cultures on hydrogels and membranes at the specified time points. Cells grown on membranes were gently scraped off with a spatula, and the harvest of two membranes was collected in PBS (−/−) (200 µL). In contrast, cells grown on scaffolds were collected together with the hydrogel scaffold in PBS (−/−) (200 µL). All samples were immediately snap-frozen in liquid nitrogen and stored at −80 °C for further analysis.

On the day of the assay, adherent cells on hydrogels were detached through a series of freeze–thaw cycles alternating between −80 and 37 °C for 5 min each with three repetitions. After this, TrypLE™ (200 µL, Thermo Fisher Scientific, Darmstadt, Germany) express enzyme was added, and samples were incubated for 5 min at 37 °C to ensure the complete detachment of the cells. Hydrogels were then carefully removed with tweezers, and the resulting cell suspensions were kept on ice for further analysis. An analogous TrypLE™ treatment was administered to cells harvested from membranes, ensuring methodological uniformity across samples. Additionally, non-seeded hydrogels in PBS (−/−) underwent TrypLE™ treatment, serving as blank controls. The latter was essential to confirm the enzymatic stability of the scaffold under the applied conditions since solubilized dSIS-MA components could falsify the mucin content. Normalization of the collected data was performed with respect to the seeding area and the DNA content (cf SM) of each sample, ensuring the accurate quantification of cell-bound mucins.

#### 2.8.3. Mucin Quantification via Periodic Acid–Schiff Base (PAS) Reaction and Alcian Blue (AB) Staining

Soluble mucins in the collected supernatants and cell-bound mucins in cell suspensions were quantified similarly according to the following procedure.

Quantification of Neutral Mucins (PAS Staining): Following our previous protocol, ref. [26] a mixture was prepared from 2% *w*/*v* CHAPS (20 µL) and either the sampled mucin-containing cell suspensions (30 µL) or supernatants (30 µL), which was further diluted with PBS (−/− 50 µL). This mixture was then sequentially incubated with periodic acid (20 µL) for 2 h and Schiff’s reagent (100 µL) for 1 h at 37 °C in a 96-well plate. The development of a pink-colored solution indicated the presence of neutral mucins. The absorbance was measured at 555 nm.

Quantification of Acidic Mucins (AB Staining): Adapted with modifications from Zamora et al. [36], either the sampled mucin-containing cell suspensions (30 µL) or supernatants (30 µL) and 2% *w*/*v* CHAPS (20 µL) were mixed and diluted with 1 M HEPES buffer (50 µL). The suspension was incubated for 2 h with a solution of 1 *w*/*v*% AB in 3% *v*/*v* acetic acid (100 µL), which was filtered (0.2 µm) before use. Following incubation, the mucin–alcian blue complex was centrifuged at 2500× *g* for 15 min at RT, leaving a pellet of blue gel. Wash and spin cycles using 40% ethanol/60% 0.1 M sodium acetate buffer with 25 mM MgCl_2_ at pH 5.8 were performed to purify the pellets, which were finally fully dissolved in 10% SDS solution for absorbance measurement at 620 nm.

Mucins from bovine submaxillary glands served to prepare a standard calibration curve. Known concentrations of mucins were prepared and analyzed identically and in parallel with experimental samples. To fall within the assay’s linear range, samples were diluted accordingly with PBS (−/−) for PAS or HEPES for AB stains.

### 2.9. Statistical Analysis

The statistical significance of the gathered data was determined using the non-parametric Kruskal–Wallis test, followed by Dunn’s multiple comparisons test using OriginPro software (Version 2021b. OriginLab Corporation, Northampton, MA, USA). *p*-values *p* < 0.05 were considered statistically significant and indicated by * or otherwise non-significant (n.s.).

## 3. Results and Discussion

### 3.1. Design of Hydrogel-Integrated Millifluidic Tissue Chamber and Flow Circuit

With the emergence of organ-on-a-chip technology, microfluidic systems have evolved as a great tool to apply fluid shear stress on cells and tissue models. The field of organoid-derived ISC research has thus far concentrated on the colon, with comparatively less attention being paid to the small intestine, especially the terminal ileum [14,37]. A limiting factor for ISC-derived small intestinal models is the challenging and sustained culture of fully confluent 2D monolayers derived from human small intestinal organoids [38,39]. The development of hydrogel-integrated microfluidic systems has facilitated the creation of a homeostatic ISC-derived mini-gut model [40]. Although microfluidic devices are highly versatile, their manufacture is not easily scalable and involves a time-consuming, multi-step process associated with substantial expenses, requiring clean room facilities. Moreover, the generated tissue inside a microfluidic channel is not easily accessible for direct manipulation or analysis via SEM or AFM.

To address these challenges, we developed a scalable and cost-efficient, 3D-printed millifluidic tissue chamber setup [26]. Initially tailored for HT29-MTX cell cultivation, we redesigned our setup to securely accommodate multiple 3D-printed hydrogel scaffolds without the risk of buoyancy. The chamber design ensures uniform laminar fluid flow, which is crucial for the application of homogenous apical shear stress in order to study ISC and epithelial cell differentiation following scaffold cellularization. The surface area for cell seeding and the medium volume within the chamber were significantly reduced in order to facilitate economic cell culture, including that of human organoid-derived ISCs. Figure 1A–D illustrate the CAD of the chamber securing nine scaffolds within size-matched cavities to apply surface shear stresses within the physiological range of the intestine (0.002–0.08 dyne/cm^2^ [41]) at defined flow rates. The fully assembled chamber, tightly sealed with a transparent lid, is shown in Figure 1E,F. CFD simulations confirmed a homogeneous laminar flow through the wide but flat flow channel. This was evidenced by the observation of uniform velocity streamlines and homogeneous shear stress on the surface of flat objects within the chamber cavities at a set flow rate of 1.42 mL/min (Figure 1G,H). CFD simulation under identical conditions, but with 3D villi-structured instead of flat objects, revealed a surprisingly homogenous flow profile with a low shear stress gradient that increased from the base to the tip of the villi, closely emulating the physiological situation (Figure S1). The chamber was 3D-printed using a simple DLP printer with an autoclavable, biocompatible, and cost-effective commercial resin, ensuring usability and affordability. The manufacturing process, which takes 45 min, requires 40 mL of resin, resulting in consumable costs of EUR 5.93 per chamber [42].

The interlinking of two chambers, a medium reservoir with gas exchange, and a peristaltic pump within a single circuit enabled continuous media circulation, resulting in the generation of a defined physiological shear stress of 0.01 dyne/cm^2^ at a set flow rate of 1.42 mL/min. The implementation of an Arduino-based pump control system enables the precise adjustment of shear stress levels within this setup from 0.005 to 0.1 dyne/cm^2^. This range can be further expanded by altering the flow channel height. The setup offers the potential for flexible experimental throughput, similar to that observed in our previously reported flow chambers [26], and therefore has utility for diverse research applications.

### 3.2. dSIS-MA-Based Hydrogel Scaffolds for Advanced Small Intestinal Models

We prepared biomimetic hydrogels mimicking the stiffness range of the intestinal extracellular matrix. This was achieved by using 1.5 wt% dSIS-MA, in accordance with our previously established procedure, with only minor adjustments [33]. First, porcine small intestinal submucosa (SIS) was decellularized, enzymatically solubilized, and functionalized with photoreactive methacrylic groups to yield dSIS-MA as a biomaterial resin for 3D printing via vat photopolymerization (Figure S2A,B). In contrast to our previous protocol [33], the enzymatic solubilization of dSIS was achieved using papain instead of pepsin, resulting in comparable material properties at a lower cost [32]. The degree of functionalization with photoreactive groups was approximately 82%, as confirmed by ^1^H NMR (Figure S2B) and a chromogenic TNBS assay (Figure S2C). Furthermore, the analysis of CD demonstrated the maintenance of protein structural integrity in the dSIS-MA biomaterial resin following chemical functionalization (Figure S2D). Standardized quality control measurements are imperative for each new dSIS-MA batch, as this ensures the consistency and reliability of the resulting material properties [43].

The fabrication of flat disk-shaped hydrogel scaffolds (*d* = 6 mm and *h* = 1 mm) was achieved via layer-by-layer 3D printing from a 1.5 wt% dSIS-MA resin formulation containing 1 wt% photoinitiator, as previously reported [33], ensuring scalability and precision (Figure S3A,B). Although our printing process allows for customization into various 3D shapes relevant to the intestine [33,44,45], we opted for flat surfaces to ensure high cell seeding efficiency and facilitate convenient downstream microscopic readout. The optimal height of 1 mm for scaffolds with a diameter of 6 mm was determined to prevent floating under flow conditions, thereby minimizing sample loss. By optimizing the curing time during printing, the stiffness range of healthy human small intestinal tissue (Young’s modulus: 1.3–4.0 kPa) [46] was matched at the given biopolymer and photoinitiator concentrations. The targeted surface stiffness was approximately 15 kPa, which has previously been demonstrated to support efficient ISC adhesion and proliferation [33]. Rheological and AFM nanoindentation analyses confirmed the reproducibility of bulk stiffness (3.7 ± 0.2 kPa) (Figure S3C) and surface stiffness (14.3 ± 8.8 kPa) (Figure S3D) across various batches, meeting the mechanical criteria for advanced intestinal tissue models. Notably, even moderate variations in the mechanical properties of synthetic hydrogels within the low kPa range (soft = 0.6 kPa, medium = 2.4 kPa, and stiff = 9.6 kPa) have been demonstrated to significantly impact the stemness and differentiation pathways of ISCs [21].

Furthermore, the scaffold’s porous microarchitecture [33] was confirmed as essential for nutrient and biochemical signal exchange during cell culture. While the wet hydrogels indicate a pore size of up to 5 µm according to the widths of the darker areas and surface cross-section in AFM topography measurements in quantitative imaging mode (Figure S3D), SEM images of the lyophilized dry samples (Figure S3E) indicate a porous interior with pore sizes up to 100–150 µm and collapsed pores on the gel surface.

### 3.3. Validation of the Millifluidic System with Mucus-Producing HT29-MTX Cells

**Figure 3:**
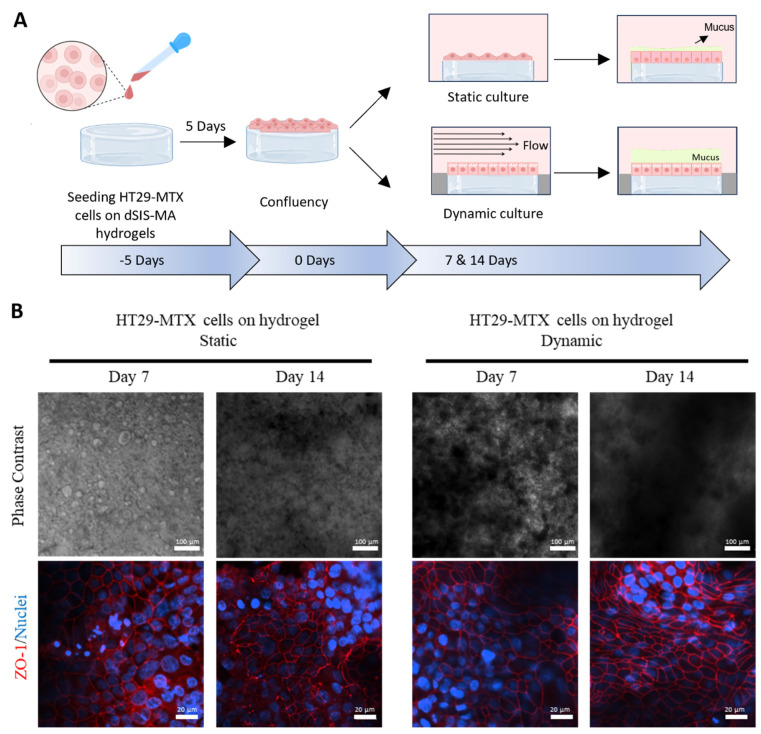
Validation of the newly designed, hydrogel-integrated flow chamber with HT29-MTX cells seeded on dSIS-MA (1.5 wt%) hydrogels under static and dynamic (shear stress: 0.01 dyne/cm^2^) culture conditions on days 7 and 14. (**A**) Illustration of the experimental workflow. (**B**) Representative phase-contrast (top) and confocal fluorescence images (bottom, in one plane) stained for the tight junction protein zonula occludens-1 (ZO-1, red) with cell nuclei counterstain (blue).

The newly designed millifluidic system was empirically validated using intestinal cell-seeded hydrogels. Given our previous findings regarding the impact of shear stress on mucus-producing HT29-MTX cells cultured on TCPS substrates [26], we utilized these cells and anticipated enhanced differentiation, mucus formation, and 3D organization of the cell monolayer under physiological shear stress. Control experiments were conducted under static conditions to isolate the effects attributable to the hydrogel scaffolds alone. The experimental setup, outlined in Figure 3A, served to validate the functionality of the millifluidic system and elucidate the influence of dSIS-MA hydrogels on cellular behavior, particularly mucus production.

Initially, HT29-MTX cells were seeded on hydrogel scaffolds and cultured statically in 96-well plates until reaching confluency. Subsequently, they were transferred to the tissue chamber for dynamic culture or maintained statically for 7 and 14 days. Phase-contrast imaging revealed distinct dark areas on day 14 under static conditions and on days 7 and 14 under dynamic conditions (Figure 3B; top row), indicating the accumulation of secreted mucus [10,14] and a 3D reorganized cellular monolayer [26]. The multilayered structure was more pronounced under dynamic culture conditions, presenting challenges for focusing. Immunofluorescence staining of the tight junction marker ZO-1 on days 7 and 14 indicated comparable epithelial barrier development on the hydrogels, regardless of shear stress (Figure 3B, bottom row).

**Figure 4:**
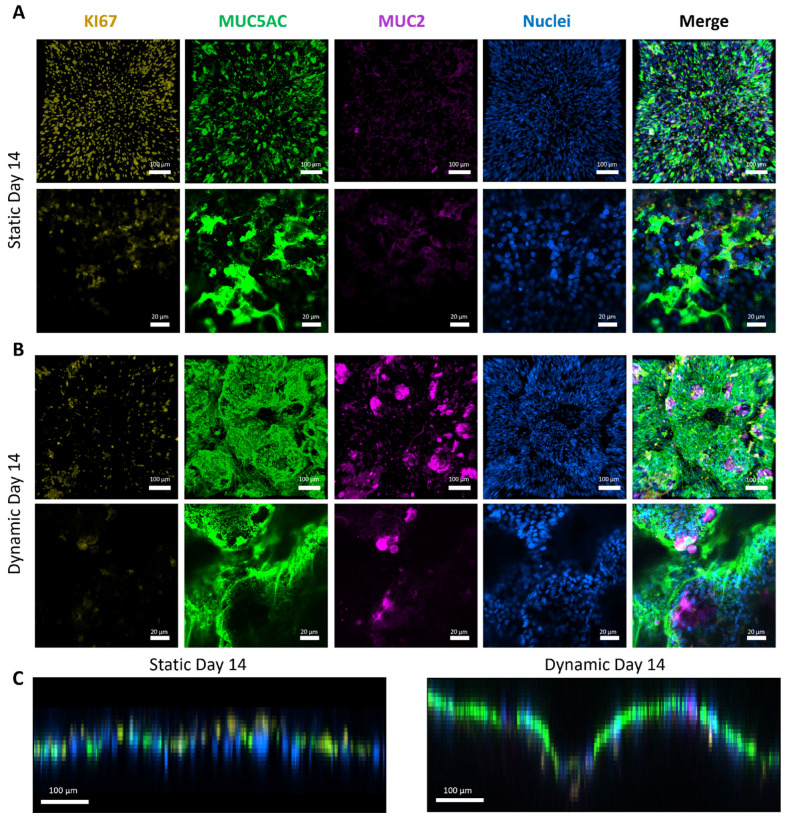
(**A**) Representative fluorescent confocal microscopy images of HT29-MTX cells cultured statically on dSIS-MA (1.5 wt%) hydrogels on day 14, stained via IF for KI-67 (yellow) to indicate proliferation and MUC5AC (green) and MUC2 (purple) as mucin markers, with nuclei counterstain (blue). Top: xy projection of z-stack images; Bottom: higher-magnification 2D images. (**B**) Representative fluorescent confocal microscopy images of HT29-MTX cells cultured under dynamic conditions (shear stress: 0.01 dyne/cm^2^), stained and imaged with identical settings to (**A**). (**C**) Side projections (xz/yz) of z-stacks from (**A**,**B**). Samples are collected from three independent runs (n = 3).

To further investigate cellular differentiation and confirm augmented mucus formation under dynamic conditions, we conducted immunofluorescence staining. Static culture controls exhibited robust proliferation on both days 7 (Figure S4A) and 14 (Figure 4A), as evidenced by the high prevalence of KI-67^+^ cells across the cell layer. Additionally, mucin detection, specifically MUC2 and MUC5AC, hardly increased from days 7 (Figure S4A) to 14 (Figure 4A), indicating no evident cellular maturation and differentiation over time under static conditions.

Remarkably, under fluid flow conditions, there was a notable reduction in proliferative KI-67^+^ cells on both days 7 (Figure S4B) and 14 (Figure 4B). This suggests that cellular differentiation into mature tissue occurred more rapidly than in static conditions. Similar observations, but on a slower timescale, were reported with HT29-MTX on hard substrates under dynamic culture, showing increased proliferation within the first two weeks of culture [10,26]. However, this proliferation becomes markedly reduced until week 3 [26]. The biomimetic properties of the dSIS-MA scaffold [33] are likely to create a microenvironment that, in conjunction with shear stress, significantly promotes cellular maturation and differentiation. This results in a notable decrease in cell proliferation as early as day 7. Consequently, there was a notable elevation in both mucin MUC2 and MUC5AC expression as early as day 7 (Figure S4B) under dynamic conditions, exceeding levels observed in statically cultured cells by day 14 (Figure 4A). This elevated mucin expression was further intensified by day 14 under dynamic conditions (Figure 4B), which is in full agreement with the phase-contrast images. Notably, hydrogels subjected to flow exhibited distinctive 3D organization of cellular layers, which was in stark contrast to their counterparts in static culture (Figure 4C and Figure S4C).

Importantly, matured tissue models under dynamic conditions showed no differences in scaffold-hosting cavity location within the chamber or chamber position within the circuit assembly. This confirms the functionality and validity of the newly developed tissue chamber for the controlled and scalable application of shear stress to cell-seeded hydrogel scaffolds.

Surprisingly, our current tissue model cultured on hydrogels demonstrated a significant enhancement in mucin MUC2 and MUC5AC expression compared to identical conditions on TCPS [26]. This underscores the synergistic effect of dynamic culture’s shear stress with dSIS-MA hydrogels’ bioactive properties, driving cell differentiation toward a goblet cell-like phenotype with increased mucus production.

### 3.4. Culture of Organoid-Derived ISCs on Scaffolds under Static and Dynamic Conditions

Next, the validated millifluidic system was challenged with human organoid-derived ISCs from the terminal ileum cultured exclusively in WRN-containing expansion media. The ISC growth and differentiation on dSIS-MA scaffolds (without transitioning to differentiation medium) under both static and dynamic conditions were examined, comparing them to conventional static cultures on Matrigel^TM^-coated insert membranes. A similar experimental design was employed for the ISC monolayers on scaffolds, as previously described for the HT29-MTX cells. The monolayers were either subjected to flow in tissue chambers or maintained statically in well plates for a duration of 5–7 days. Monolayers cultured statically on insert membranes served as controls. RNA isolation for RT-qPCR analysis was conducted after 3 days of culture, while samples were fixed for immunofluorescence (IF) staining directly after reaching confluence or on days 5–7. A time-dependent assessment of ALP activity and mucus quantification, both in the culture medium and attached to the tissue, was conducted on days 0, 3, 5, and 7.

#### 3.4.1. Formation and Culture of ISC-Based Monolayers on Scaffolds

**Figure 5:**
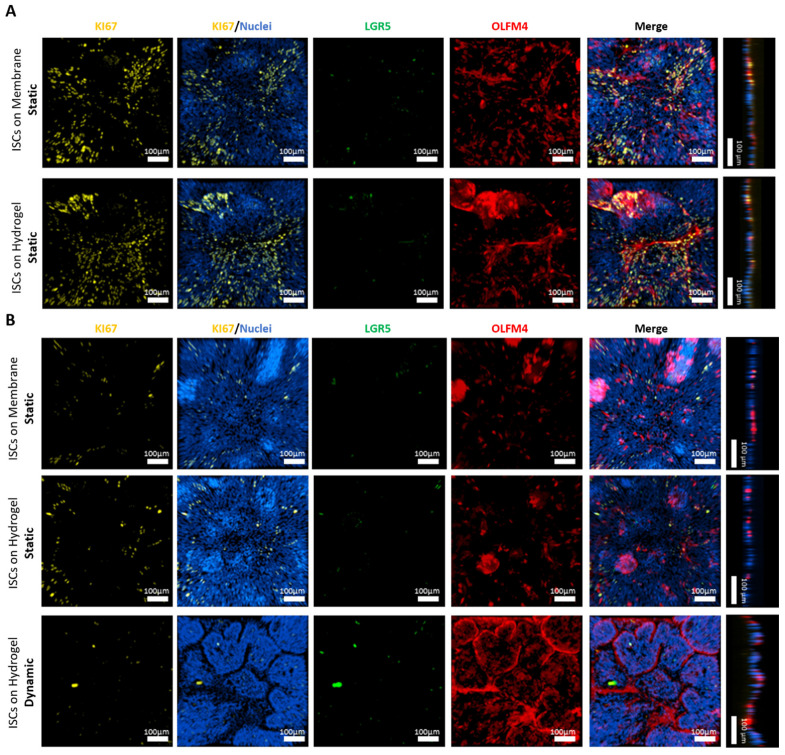
Transfer of the experimental approach to undifferentiated organoid-derived ISCs from the human terminal ileum cultured in expansion media. (**A**) Representative confocal fluorescent images of ISC monolayers cultured in static conditions on Matrigel^TM^-coated insert membranes and hydrogels at the onset of confluency (Day 0). IF staining for proliferative marker KI-67 (yellow), stem cell marker LGR5 (green), and progenitor cell marker OLFM4 (red) with nuclei counterstain (blue). For fluorescent staining, samples are collected from three independent runs (n = 3) and presented as xy projections of their z-stacks, along with their xz/yz projections. (**B**) Representative IF images of ISCs on days 5–7 cultured in expansion media under static and dynamic conditions, stained for the same markers used in (**A**). For fluorescent staining, samples are collected from three independent runs (n = 3) and presented as xy projections of their z-stacks, along with their xz/yz projections.

The ISCs were cultured on hydrogels and membranes for 2–3 days following their dissociation into single cells and small cell clusters of approximately ten cells. The seeding of ISCs at a high cell density under these conditions facilitated the rapid formation of intact and confluent cell monolayers on the scaffolds, exhibiting minimal irregularities similar to those observed on Matrigel^TM^-coated membranes (Figure S5A). IF analysis of successfully generated flat monolayers after reaching confluency (day 0) revealed the presence of highly proliferative KI-67^+^ cells distributed randomly throughout the layers both on membranes and hydrogels (Figure 5A).

A markedly higher expression of the progenitor cell marker olfactomedin 4 (OLFM4) compared to the stem cell marker leucine-rich repeat-containing G protein-coupled receptor 5 (LGR5) suggests a confluency-triggered shift from stem cells toward more mature epithelial progenitor cells. However, the relatively low IF staining efficiency of the LGR5 antibody needs to be considered. This is due to the fact that the LGR5 protein is expressed at low levels in native intestinal tissue and ISC-based models [47]. Following a period of 5–7 days in static conditions (Figure 5B), a moderate decrease in KI-67^+^ and OLFM4^+^ cells was observed on both substrates in comparison to day 0, indicating a continual process of maturation. This observation is consistent with the findings of Van Dussen et al., who reported a pronounced decrease in *MKI67* and *OLFM4* mRNA expression levels in human ileal organoids following chemically induced differentiation (removal of WRN from the medium) [19].

Under dynamic conditions, a notable decrease in KI-67 and an increase in OLFM4 expression were observed in comparison to day 0, with stable LGR5 levels across all conditions (Figure 5B). This finding was corroborated by an RT-qPCR analysis of cells on hydrogels three days post-confluency, which revealed a moderate 2.5-fold increase in *LGR5* and a pronounced, nearly 20-fold increase in *OLFM4* under dynamic compared to static conditions (Figure S6A). Furthermore, an increase in *OLFM4* mRNA levels was observed in colon-derived ISCs cultured on-chip for eight days under dynamic conditions [15]. Interestingly, OLFM4 protein expression in ISCs was accompanied by a decrease in LGR5 when the substrate stiffness was increased from 0.6 to 2.4 kPa in static culture [21], a stiffness comparable to that of the hydrogels used in this study (3.7 kPA). The precise roles of OLFM4 in ISC differentiation in health and disease remain incompletely understood [48]. Following a period of 5–7 days in expansion media, ISCs subjected to shear stress organized into 3D villi-like protrusions (Figure 5B and Figure S7A), as observed with HT29-MTX cells on hydrogels (Figure 4C and Figure S4C) and TCPS substrates [26], resulting in zonated areas with high OLFM4 expression at their boundaries in the case of the ISC culture. Similar 3D protrusions have been observed with ISCs from the human colon and duodenum when cultured on-chip under physiological shear stress [14,39]. Despite the provision of essential stem cell niche factors (WRN) in our expansion media, a decline in active ISC proliferation during 2D culture was noted, particularly under dynamic conditions. This is likely due to the progression of cellular differentiation induced by mechanical stimulation, as observed in ISC-based colon-on-chip [14] and jejunum-on-chip models [49].

It is important to note that throughout the course of this study, numerous unsuccessful cell seeding attempts were conducted, resulting in the formation of holes in the monolayers. These instances constituted approximately 40% of all trials and were therefore excluded from further analysis (Figure S5B). Similar failure rates (approximately 60%) have been reported with human small intestine-derived stem cells, which were improved using fragmented organoids instead of single cells [39]. The challenges in reproducing fully confluent, intact monolayers of human ISCs derived from the terminal ileum are currently being addressed in ongoing research.

Additionally, under physiological shear stress, three morphological categories of monolayers were observed, with the majority featuring intact villus-like structures adhering tightly to the scaffold (Figure S7A and Figure 5B). However, starting on day 7, monolayers exhibited villi displacement, likely due to flow dynamics and rapid in vivo-like intestinal cell turnover (Figure S7B). Consequently, by day 14 of dynamic culture, only a few dispersed cells remained on the scaffolds, as similarly described in the literature [21] and attributed to insufficient stem cell preservation, currently limiting the experimentation time of our model to around 5 days. Some cases revealed flatter monolayers devoid of protrusions with pore-like patterns upon ZO-1 staining (Figure S7C). This observation might be a result of shear stress-induced noncanonical autophagy, evident by the formation of 10–20 µm vacuoles in intestinal monolayers, as reported previously [50], and requires further investigation. The observed morphological variability suggests varying degrees of cellular differentiation between samples, with some potentially shedding previously formed 3D protrusions in response to shear forces. For future studies, it is recommended that dynamically cultured samples are collected on day 5 rather than day 7 and that interval exposure to shear stress is employed to better mimic the physiological situation in the human intestine, allowing for tissue regeneration during static resting phases.

#### 3.4.2. Differentiation of ISC-Based Monolayers and Tissue Formation

Expanding on the transformative impact of shear stress from stemness toward differentiation, we examined structural, functional, and differentiation markers of ileal ISC monolayers. Following 5–7 days of culture in expansion media, immunofluorescence (IF) staining of F-actin, E-cadherin (E-cad), and zonula occludens (ZO-1) protein markers revealed an intact cytoskeletal architecture and the presence of adherens and tight junctions across all samples and conditions (Figure S8). These confirmed epithelial features are essential for epithelial integrity and barrier function.

**Figure 6:**
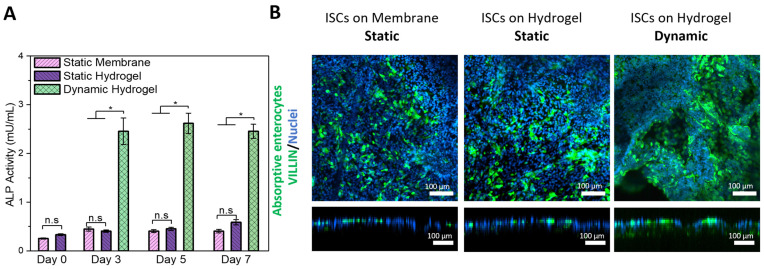
Mechanically stimulated differentiation of human terminal ileum-derived ISCs into functional enterocytes by physiological shear stress exposure. ISCs were cultured on insert membranes and dSIS-MA hydrogels (1.5 wt%) statically or dynamically in expansion media for 5–7 days after reaching confluency. (**A**) ALP activity was assessed on days 0, 3, 5, and 7 post-confluency from supernatants of ISC monolayers on a culture area of around 3.4 cm^2^. Data are presented as mean ± SD for n = 3, where “*” indicates statistical significance (*p* < 0.05) and “n.s.” indicates non-significance according to the Kruskal–Wallis test, followed by Dunn’s test. (**B**) Representative confocal images in xy projections and their xz/yz projections of acquired z-stacks after IF staining of villin (green), indicating absorptive enterocyte with counterstained nuclei (blue), on days 5–7 cultured statically or dynamically on insert membranes and hydrogels in expansion media.

Next, we investigated the potential for the multi-lineage differentiation of ISCs cultured under dynamic conditions to generate cells characteristic of enterocytic, enteroendocrine, Paneth, and goblet cell lineages. Shear stress augmented ISC differentiation into the enterocyte lineage compared to static cultures, as evidenced by the increased mRNA expression of alkaline phosphatase (*ALPI*, ~30-fold) and sucrase-isomaltase (*SI*, ~18-fold) on day 3 of culture (Figure S6B). This was further supported by a significant rise in extracellular alkaline phosphatase (ALP) activity in supernatants on days 3, 5, and 7 of dynamic compared to static cultures, irrespective of the culture substrate (Figure 6A). Additional immunofluorescence staining of the enterocyte protein marker villin on samples collected after 5–7 days of culture confirmed the findings from RT-qPCR and ALP activity assays (Figure 6B), as well as cell polarization. Intriguingly, in the absence of chemical differentiation inducers, shear stress alone can mechanically stimulate functional ISC differentiation into the absorptive enterocyte lineage, which is shown here for the first time on the enzymatic activity level. Functionally differentiated and matured enterocytes play a crucial role in intestinal models, emulating primary functions such as nutrient absorption and barrier maintenance.

**Figure 7:**
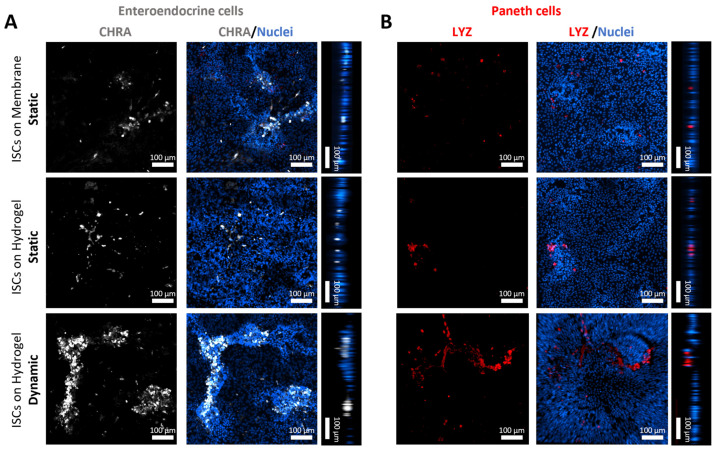
Representative z-stacks of confocal fluorescence microscopy images presented as xy projections and their xz/yz projections. Protein expression of (**A**) chromogranin A (white) and (**B**) lysozyme (red) in ISC-based monolayers with counterstained nuclei (blue) on days 5–7 cultured statically or dynamically on insert membranes and hydrogels in expansion media. Samples are collected from three independent runs (n = 3).

Further RT-qPCR analysis assessed on day 3 revealed that shear stress resulted in a four- and fivefold upregulation of chromogranin A (*CHRA*) and lysosome (*LYZ*) expression in ISCs, respectively, in comparison to static culture on dSIS-MA hydrogels (Figure S6C). The results indicate that the ISC can be effectively differentiated into the enteroendocrine and Paneth cell lineages. This is further substantiated by IF staining of the expressed protein markers (Figure 7A,B). Particularly under dynamic conditions, enteroendocrine-like cells localize at positions of the generated 3D protrusions, while Paneth-like cells, at least under shear stress, appear at the bottom of the cellular layer. This is similar to what is observed in vivo, where they are located in close proximity to the crypts. Enteroendocrine and Paneth cells are integral components of the ISC niche, and their presence is crucial for maintaining a functional niche and ISC homeostasis.

Our findings are consistent with previous research on human small intestinal stem cells subjected to flow conditions, particularly regarding increased markers for absorptive enterocytes and enteroendocrine cells. However, discrepancies arise concerning Paneth cells. Kasendra et al. observed enhanced differentiation of human duodenum-derived ISCs after switching to a differentiation medium lacking WRN within a microfluidic environment. This was accompanied by increased transcriptional levels of markers for absorptive enterocytes and enteroendocrine cells, alongside decreased Paneth cell markers [39,51]. The precise mechanism by which these changes occur remains unclear. It is possible that they result solely from mechanical stimulation, the transition to differentiation medium, or a combination of both. Yin et al. found that shear stress on-chip alone induced the differentiation of human jejunum-derived ISCs into absorptive enterocytes and enteroendocrine cells when cultured in expansion media, which is consistent with the findings of our study [49]. However, Paneth cell markers were not assessed for the jejunal model, while none of the above studies confirmed functional absorptive enterocyte differentiation as demonstrated by ALP production and secretion in our study. Our results suggest a unique synergistic effect of shear stress and the dSIS hydrogel environment on ISC differentiation toward the Paneth cell lineage requiring more in-depth investigations. A comprehensive gene sequencing study on ISCs cultured on our hydrogel scaffolds comparing static and dynamic conditions is currently in progress.

#### 3.4.3. Mucus Production and Secretion of ISC-Based Monolayers on Scaffolds

**Figure 8:**
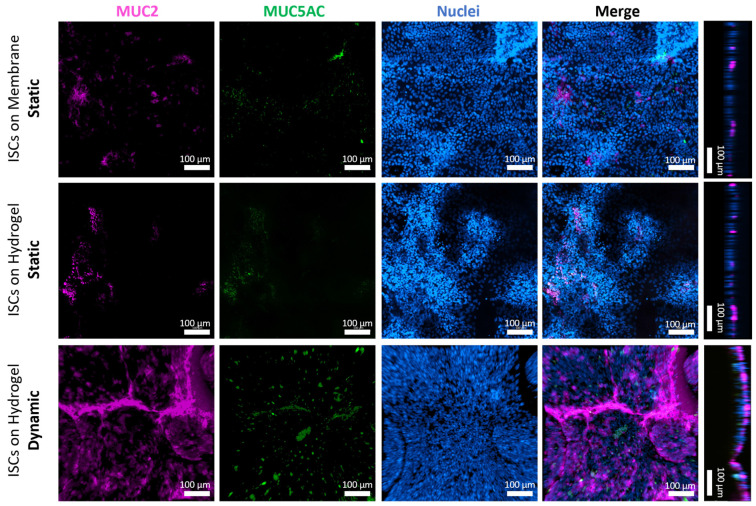
Representative confocal z-stack images in xy projection, along with their xz/yz projections, of ISCs stained for the MUC2 (purple) and MUC5AC (green) and counterstained with nuclei (blue) after static culture on insert membranes and hydrogels or dynamic culture on hydrogels on days 5–7 in expansion media. Samples are collected from three independent runs (n = 3).

Goblet cells play a crucial role in the intestinal epithelium by producing mucins, particularly MUC2. Quantitative RT-PCR analysis on day 3 revealed the strong upregulation of gel-forming mucins *MUC2*, *MUC5AC*, and *MUC5B*, as well as the moderate upregulation of *MUC17* and trefoil factor 3 (*TFF3*) in ISCs cultured on dSIS-MA (1.5 wt%) hydrogels under shear stress conditions, compared to static culture (Figure S6D). IF staining on days 5–7 confirmed a markedly increased production and secretion of MUC2 and MUC5AC in cells cultured dynamically on hydrogels, compared to basement levels through spontaneous cell differentiation observed in static controls, all maintained in expansion media (Figure 8). Notably, MUC2 protein expression levels surpass MUC5AC levels, while the opposite is observed for their transcriptional levels.

Similarly to a duodenum-on-chip study, in which increased *MUC2* and *MUC5AC* mRNA levels were detected upon chemically induced differentiation [39], our findings show the same effect solely via mechanical stimulation without chemical differentiation. Conversely, in a colon-on-chip setup, *MUC2* mRNA levels increased while *MUC5AC* decreased after eight days of culture under flow in expansion media, compared to static conditions [15]. The discrepancy in *MUC5AC* expression highlights the complex relationship between mucin expression and intestinal health. Increased *MUC5AC* expression, typically associated with the gastric and respiratory tracts, may indicate a protective or adaptive response to stress or injury in the small intestine [52]. This suggests further investigation into the mechanisms and implications of this response for disease progression or healing.

To further characterize the secreted mucus within our experimental setup, quantitative assays employing alcian blue (AB) staining for an acidic and periodic acid–Schiff base reaction (PAS) for neutral mucins were utilized. However, the direct staining of acidic and neutral mucins is not possible when dSIS-based hydrogels are used as a culture substrate due to significant background staining of the gels’ ECM components (Figure S9). Therefore, a modified protocol was developed, in which the cellular layer is first removed from the scaffolds before assessing acidic and neutral mucins.

**Figure 9:**
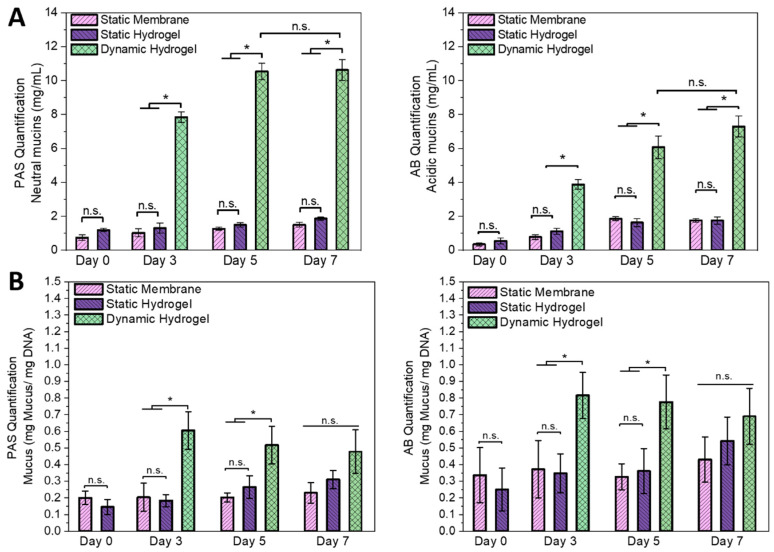
Time-dependent quantification of neutral (PAS assay) and acidic (AB assay) mucins produced by ISCs during static and dynamic culture on Matrigel^TM^-coated insert membranes and dSIS-MA (1.5 wt%) hydrogels for estimation of the amount of secreted mucus via a standard calibration curve using bovine submaxillary gland mucins. (**A**) Amount of mucins shed to the culture media by intestinal monolayers on a substrate area of approximately 3.4 cm^2^. Samples were taken from the apical supernatant in static cultures and the media reservoir in dynamic cultures and normalized accordingly. (**B**) Amount of cell- or membrane-bound mucins normalized to the DNA content of the monolayer. Data are presented as mean ± SD for independent runs n = 3, where “*” indicates statistical significance (*p* < 0.05) and “n.s.” indicates non-significance of all samples performed by the Kruskal–Wallis test, followed by Dunn’s test.

Our assessment of the loose shed mucus both in the apical medium compartment of static cultures and in the medium reservoir of dynamic cultures (Figure 9A), as well as the cell-attached mucus (Figure 9B) of both conditions on specified days, revealed a distinct pattern. Significantly larger amounts of mucus were secreted under dynamic compared to static conditions, which is in line with enhanced ISC differentiation into functional, mucin-producing goblet cells under shear stress (Figure 8). A general correlation between AB/PAS staining and mucin protein expression levels has been demonstrated in the literature [53]. The shedding of mucins from the polarized epithelial monolayers emulates the dynamic nature of the intestinal mucus layer in vivo. This layer undergoes continuous replenishment by goblet cell secretions and dispersion through peristalsis and intestinal flow, ensuring the removal of pathogens and debris while maintaining a protective barrier and facilitating nutrient uptake.

The shedding and accumulation of mucus in cell culture medium, particularly under flow conditions, was also reported with an ISC-derived ileum-on-chip model, reaching levels of around 400 µg/mL of secreted mucus in the medium after seven days of dynamic flow in expansion media, as determined using an AB assay [54]. Unfortunately, the absolute mucin concentrations are not comparable in such studies due to the varying setups affecting the cell number, medium volume in the circuit, and the exact flow rate. It is important to note that PBS (−/−) destabilizes the forming AB–mucin complex, resulting in significant complex precipitation during a quantitative AB assay. Replacing PBS with HEPES buffer resolves this issue by enhancing the stability and solubility of the AB dye and facilitating its interaction with mucins in an entropy-driven process, resulting in a more stable and soluble mucin–AB complex for subsequent absorbance-based quantification.

Technical limitations concerning the working distance in our microscopy setup did not allow for the direct assessment of mucus thickness in the unfixed state via fluorescent beads [26] due to the hydrogels’ thickness (1 mm). We are currently developing solutions to overcome these obstacles in future investigations by reducing the thickness of the hydrogels while increasing their weight through fixation on glass coverslips to prevent floating.

## 4. Conclusions

The objective of this study was to develop a hydrogel-integrated millifluidic system for the application of defined shear stress to diverse tissue models, with a particular focus on intestinal models. The designed tissue chamber is capable of accommodating nine flat or 3D-structured hydrogel scaffolds while maintaining a laminar flow profile. The chamber’s performance was initially validated with HT29-MTX cells and then challenged during the culture of human ISCs derived from the terminal ileum. Our findings demonstrate the beneficial mucus-related effects achievable during the multi-lineage differentiation and 3D structural organization of human ISCs when cultured on biomimicking hydrogel scaffolds under physiological shear stress without distinct chemical differentiation triggers. Functional differentiation into enterocyte and goblet cell lineages was confirmed via quantitative ALP activity and mucus measurements. No marked differences were observed at the protein level between Matrigel^TM^-coated and dSIS-MA hydrogel controls when culturing ISCs statically in expansion media, clearly assigning the observed cellular effects to shear stress and flow conditions. However, distinct differences were noted in HT29-MTX cells cultured dynamically on TCPS and hydrogels, highlighting the bioinstructive properties of the scaffold. Future investigations will explore the impact of the mechanical properties of culture scaffolds/substrates, accompanied by a comprehensive mRNA gene expression analysis, to reveal intricate interactions between hydrogel and ISCs. We propose that dSIS-MA hydrogels, with tunable stiffness and bioactive composition, could offer biomimetic alternatives to synthetic scaffolds [21] for modeling diseased states, such as inflammatory bowel disease [55]. Additionally, variations in medium composition will be explored to understand their influence on ISC differentiation, cellular responses, and mucus properties.

While colon-on-chip systems have achieved notable success in creating in vitro mucosal models featuring physiological mucus properties [14], analogous models of the small intestine are rare. A unique feature of our dynamic small intestinal model is the fact that it does not require co-cultured endothelial cells for proper tissue maturation, as frequently observed with gut-on-chip systems [39,51,54], allowing the investigation of isolated epithelial cell-derived effects.

The printed millifluidic chamber presents a cost-effective alternative to fluidic chip systems, enabling flexible design modifications to suit diverse experimental requirements and boost sample throughput. This work opens avenues for elucidating complex interactions within the intestinal epithelium under dynamic conditions, with the resulting tissue models providing promising avenues for advanced disease modeling, drug discovery, and pathogen studies. Future work will address challenges such as the limited self-sustainment of ISC-based tissue models under shear stress by including more physiologically relevant features, such as crypts, which will protect ISCs regionally from shear stress exposure.

## Data Availability

Data are reported within the article and in the Supplementary Materials. Further data will be made available through the corresponding author upon reasonable request.

## Supplementary Materials

The following supporting information can be downloaded at: https://www.mdpi.com/article/10.3390/cells13131080/s1, Table S1: Antibodies used in IF staining; Table S2: Primers used in RT-qPCR analysis; Figure S1: CFD simulation of 3D villi-structured hydrogels in the millifluidic tissue chamber showing shear stress gradient along the villi. Figure S2: Processing, modification, and characterization of dSIS-MA biomaterial resins; Figure S3: Characterization of printed dSIS-MA hydrogels; Figure S4: Confocal images of HT29-MTX under static and dynamic conditions on day 7 showing KI-67, MUC2, and MUC5AC expression; Figure S5: Phase-contrast images of human organoid-derived ISC monolayers under static conditions on day 0 post-confluency; Figure S6: Quantitative RT-qPCR analysis of ISCs on hydrogels on day 3 post-confluency; Figure S7: Confocal images of ISC monolayers under dynamic conditions on days 5–7 post-confluency; Figure S8: Confocal images of ISCs under static or dynamic conditions showing F-actin, E-cadherin, and ZO-1; Figure S9: 3D-printed hydrogel scaffolds showing background staining of AB and PAS after water washes; Table S3: Jumper pins configuration of the X,Y,Z, and A axes for the step motors; Figure S10: settings for different configuration modes of standard micro-stepping and a photograph of the operational quarter step mode set on Arduino board; Figure S11: Step-by-step assembly of the Arduino-controlled pump system. References [32,33,43,56] are cited in the Supplementary Materials.
