# Supplementary material for: Hydrogel-Integrated Millifluidic Systems: Advancing the Fabrication of Mucus-Producing Human Intestinal Models
Source: Cells. 2024 Jun 21;13(13):1080. doi: 10.3390/cells13131080 (PMC11240340; doi:10.3390/cells13131080)
Supplement: Supplementary file 1 [file cells-13-01080-s001.zip › Almalla et al_Cells_SI.pdf]

Hydrogel-Integrated Millifluidic Systems: Advancing the Fabrication of Mucus-producing Human Intestinal Models

Ahed Almalla,[1] Nadra Alzain,[1,2] Laura Elomaa,[1] Fiona Richter,[1] Johanna Scholz,[1] Marcus Lindner,[1] Britta Siegmund,[2] Marie Weinhart[1,3,]*

[1]*Institute of Chemistry and Biochemistry, Freie Universität Berlin, Takustr. 3, 14195 Berlin, Germany*

[2]*Department of Gastroenterology, Infectious Diseases and Rheumatology (including Nutrition Medicine), Charité Universitätsmedizin Berlin, corporate member of Freie Universität Berlin and Humboldt-Universität zu Berlin, Hindenburgdamm 30, 12203 Berlin, Germany*

[3]*Institute of Physical Chemistry and Electrochemistry, Leibniz Universität Hannover, Callinstr. 3A, 30167 Hannover, Germany*

* Corresponding author: marie.weinhart@fu-berlin.de and marie.weinhart@pci.uni-hannover.de

Table of Contents

1. Materials and Methods 2
   1.1. Materials and Reagents 2
   1.2. Decellularization, Solubilization, and Methacrylation of Porcine Small Intestinal Submucosa (SIS) 5
   1.3. 3D printing of dSIS-MA-based Hydrogels 6
   1.4. Tissue Chamber Accessory 6
2. Material Characterization 7
   2.1. dSIS-MA Resin Characterization 7
   2.2. Rheology of the dSIS-MA-based Hydrogels 8
   2.3. Atomic Force Microscopy (AFM) and Scanning Electron Microscopy (SEM) of dSIS-MA-based hydrogels 8
   2.4. Human Crypt Isolation and Organoid Culture 9
   2.5. Real-time Quantitative Polymerase Chain Reaction (RT-qPCR) Analysis 10
   2.6. DNA Content Quantification 11
   2.7. Immunofluorescence Staining 11
3. Supplementary Tables and Figures 12
4. Controller-Board Assembly and Pump Programming 21
   4.1. Assembly of the Arduino Board to Control the Pump System 21
   4.2. Arduino Software and Programming 23

Supporting Information

1. Materials and Methods
    1.1. Materials and Reagents

Materials for dSIS-MA preparation, analysis and scaffold printing: Porcine small intestinal submucosa (SIS) was purchased from a local meat market (Rasch GmbH, Berliner Fleischgroßmarkt, Germany). Papain from *Carica papaya* (#1.07144, 30000 U/mg), sodium dodecyl sulfate (SDS, #8.22050), triton™ X-100 (#X100), methacrylic anhydride (MA, #276685), 2,4,6-trinitrobenzene sulfonic acid (TNBS, #P2297), peracetic acid (PAA; 38-40% #107222), penicillin/streptomycin (p/s, #P4333, 10,000 units penicillin and 10 mg/mL streptomycin; 100x concentrated), phosphate-buffered saline (#P4417, tablets without calcium and magnesium ions (PBS (-/-)), ethylenediamine tetraacetic acid disodium salt (EDTA-Na_2, #E4884), and L-cysteine (#168149) were purchased from Sigma-Aldrich (Sigma Aldrich/Merck KGaA, Darmstadt, Germany). Hydrochloric acid (HCl, 37%, #10244100), glacial acetic acid (>99.7%, #AA36289AE), and sodium hydroxide (NaOH, ≥ 98%, #11438327) was purchased from Thermo Fisher Scientific (Darmstadt, Germany). Lithium-phenyl-2,4,6-trimethylbenzoylphosphinat as the photoinitiator for dSIS-MA printing was synthesized according to literature [1].

Materials for tissue chamber fabrication and dynamic cell culture: Heat-seal sterilization bags (400 mm× 250 mm, SÜDPACK Medica, Baar, Switzerland), syringe filters (Minisart®, PTFE, 0.2 µm, #SRP15, Sartorius, Göttingen, Germany).

Materials for HT29-MTX culture, cell staining and fixation, and mucin quantification: Fetal bovine serum (FBS, Superior, #S0615), nonessential amino acids (MEM-NEAA), alcian blue powder (#A5268), sodium acetate (#S-2889), magnesium chloride (#M8266), periodic acid Schiff (PAS) staining kit (#395B), bovine submaxillary glands mucin (#M3895) and phalloidin–Atto® 647N (#65906), Triton X-100 (#X100), and Tween-20 (#P9416) were purchased from Sigma-Aldrich (Sigma Aldrich/Merck KGaA, Darmstadt, Germany). 3-[(3-Cholamidopropyl)-dimethyl-ammonio]-1-propansulfonate (CHAPS, #1.11662.0001) was purchased from VWR International (Darmstadt, Germany). Hoechst dye (#33342) was purchased from Thermo Fisher Scientific (Darmstadt, Germany). Gibco™ Dulbecco's Modified Eagle's Medium (DMEM, high glucose, GlutaMAX™, #35050-061) Gibco™ trypsin-ethylenediamine tetraacetic acid (Trypsin-

EDTA, #15400-054) was purchased from Thermo Fisher Scientific (Darmstadt, Germany). Mounting medium (ProTaqs® Mount Flour) was purchased from Biocyc (Luckenwalde, Germany). Paraformaldehyde (PFA, ROTI®Histofix 4%, #P087.4) and albumin fraction V (BSA, biotin-free, # 0163.4) were ordered from Carl Roth (Karlsruhe, Germany). Primary and secondary antibodies for immunofluorescence staining are listed in Table 1.

Materials for organoid isolation and culture: D-sorbitol (#6213.1, Carl Roth, Karlsruhe, Germany), sucrose (#A15583.0C, Thermo Fisher, Darmstadt, Germany), dithiothreitol (DTT) (#6908.2, Carl Roth, Karlsruhe, Germany), ciprofloxacin (#J01MA02, Fresenius Kabi, Bad Homburg vor der Höhe, Germany), fluconazole (#603231, B. Braun SE, Melsungen, Germany), primocin (#ant-pm-05, Invivogen, San Diego, CA, USA), gentamycin (#15750060, Thermo Fisher, Darmstadt, Germany), L-WRN cells (CRL-3276TM, ATCC®, Manassas, Virginia, USA), advanced Dulbecco's Modified Eagle Medium (DMEM)/F12 (#12634010, Thermo Fisher Scientific, Darmstadt, Germany), GlutaMAX™ L-glutamine (#35050-038, ThermoFisher Scientific, Darmstadt, Germany), p/s (#A2213, Merck Biochrom, Darmstadt, Germany), HEPES buffer (#15630-056, Thermo Fisher Scientific, Darmstadt, Germany), FCS (#12604013, Merck, Darmstadt, Germany), recombinant human EGF (#AF-100-15, PeproTech®, Thermo Fisher Scientific, Darmstadt, Germany), nicotinamide (#N0636, Sigma-Aldrich/Merck KGaA, Darmstadt, Germany), A 83-01 (#SML0788, Sigma Aldrich/Merck KGaA, Darmstadt, Germany), SB 202190 (#S7067, Sigma Aldrich/Merck KGaA, Darmstadt, Germany), Y-27632 dihydrochloride (#M1817, AbMole Bioscience via Hölzel Diagnostika Handels GmbH, Köln, Germany), [Leu^{15}]-Gastrin I human (#G9145, Sigma Aldrich/Merck KGaA, Darmstadt, Germany), Gibco™ TrypLE™ Express solution (#12604013: 1x from Thermo Fisher Scientific, Darmstadt, Germany), Falcon® permeable support Transwells fitting 24-well plates with a 0.4 µm transparent PET membrane (#353095, Merck KGaA, Darmstadt, Germany), Corning® Matrigel™ growth factor reduced basement membrane matrix (#CLS356231, Merck KGaA, Darmstadt, Germany).

Chelating buffer composition for crypt isolation: 54.9 mM D-sorbitol, 43.4 mM sucrose, 10 mM EDTA, 1 mM DTT, 10 µg/ml ciprofloxacin, 1 µg/ml fluconazole, 100 µg/ml primocin, and 50 µg/ml gentamycin.

Basal medium composition for crypt isolation: Gibco™ advanced Dulbecco's Modified Eagle Medium supplemented with 2 mM Corning™ cellgro™ L-glutamine, 1x p/s, and 10 mM Gibco™ HEPES.

Primary culture medium (PCM): Gibco™ advanced Dulbecco's Modified Eagle Medium supplemented with 2 mM Corning™ cellgro™ L-glutamine, 1x p/s and 20% FCS (#12604013, Merck, Darmstadt, Germany).

Expansion medium: WRN-conditioned medium mixed in a 1:1 v/v ratio with PCM supplemented with recombinant human epidermal growth factor (EGF, 50 ng/ml), nicotinamide (10 mM), A83-01 (0.5 µM), SB202190 (10 µM), and human gastrin I (10 nM) to the specified final concentrations.

Growth factor and small molecule stocks: recombinant human EGF was prepared at 100 µg/mL in 0.1% BSA/PBS (-/-). Nicotinamide was prepared at 1 M in PBS (-/-). The transforming growth factor and kinase-type-1-receptor inhibitor A 83-01 (3-(6-methyl-2-pyridinyl)-*N*-phenyl-4-(4-quinolinyl)-1H-pyrazol-1-carbothioamid) was prepared at 5 mM in DMSO. The p38-MAP-kinase inhibitor SB 202190 (4-(4-fluorophenyl)-2-(4-hydroxyphenyl)-5-(4-pyridyl)-1H-imidazole) was prepared at 30 mM in DMSO. Rho kinase inhibitor Y-27632 dihydrochloride was prepared at 10 mM in PBS (-/-). [Leu^{15}]-Gastrin I human was prepared at 0.1 mM in 0.1% NaOH.

Materials for RNA isolation and qPCR: TRIzol™ reagent (Invitrogen™, Thermo Fisher Scientific, Darmstadt, Germany), Direct-zol RNA Microprep kit (#R2062, Zymo Research, Freiburg, Germany), Amplification Grade DNase I; 100U (1U/µl) (#18068-015, including 25 mM EDTA and 10X DNase I reaction buffer, Invitrogen™, Thermo Fisher Scientific, Darmstadt, Germany) , EDTA (Thermo Fisher Scientific, Darmstadt, Germany), oligo$(dT)_{12-18}$ primers (Invitrogen™, Thermo Fisher Scientific, Darmstadt, Germany), deoxy nucleoside triphosphates (dNTPs, ROTI®Mix PCR3, Carl Roth, Karlsruhe, Germany), RNaseOUT™ recombinant ribonuclease inhibitor (20 U, Invitrogen™, Thermo Fisher Scientific, Darmstadt, Germany), DTT (Thermo Fisher Scientific, Darmstadt, Germany), and M-MLV reverse transcriptase (250 U, Invitrogen™, Thermo Fisher Scientific, Darmstadt, Germany), Aqua™ RNAse-free water (B. Braun, Melsungen, Germany), SYBR™ Green PCR Master Mix (Invitrogen™, Thermo Fisher Scientific, Darmstadt, Germany), MicroAmp™ fast optical 96-well reaction plate (Applied Biosystems, Thermo Fisher Scientific, Darmstadt, Germany), Quant-iT™ PicoGreen™ dsDNA assay kit

(Thermo Fisher Scientific, Darmstadt, Germany). Primers listed in Table 2 were ordered from TIB Molbiol Syntheselabor GmbH (Berlin, Germany).

Materials for ISC-derived monolayer staining and proliferation: Alkaline phosphatase assay kit (#ab83371) was ordered from Abcam, Regensburg, Germany. Cell fixation, staining, and mucin quantification reagents are the same as listed for HT29-MTX cultures.

1.2. Decellularization, Solubilization, and Methacrylation of Porcine Small Intestinal Submucosa (SIS)

Porcine small intestinal submucosa (SIS) stored in salt solution was decellularized according to our previously published protocol, [2] with minor modifications. Briefly, SIS was sectioned into pieces (3-4 cm) and placed in 2 L beakers, stirred for 24 hours at 4 °C in deionized water (2-3 water changes) to eliminate residual salts. Subsequently, the tissue was treated with SDS solution (1%) for 2 hours, exchanging the SDS solution once, then with Triton X-100 (1 %) for 24 hours (2-3 triton changes). The decellularized SIS (dSIS) was then extensively rinsed with water for 30 minutes, defatted in pure ethanol for 30 minutes, and sterilized in 0.1% PAA for 20 minutes. Final removal of residual chemicals was achieved through sequential washes in PBS (-/-) and deionized water over 3-5 days with 2-3 daily media changes. Following decellularization, the enzymatic solubilization of dSIS was achieved using papain, adhering to a published protocol with minor adjustments [3]. Briefly, dry and milled dSIS pieces (2 g) were incubated in 200 mL papain solution (200 mg papain in 20 mM EDTA containing 5 mM L-cysteine at pH = 5.5) at RT for 48 hours under periodic homogenization using a T 10 basic Ultra-Turrax (IKA®-Werke GmbH & Co. KG). The solubilization process was halted by adjusting the pH to 9.5-10 with a 10 M NaOH solution on ice for 1 hour to deactivate the enzyme. The solution was again homogenized and centrifuged at 140 x g for 5 minutes to discard any undigested remnants if present, and the supernatant was directly subjected to methacrylation without further processing [2]. In brief, MA (1 mL of MA per 1 g papain-treated dSIS) was incrementally added to the solubilized dSIS-containing supernatant under vigorous stirring and protected from light over a time course of 3 hours, while maintaining the pH $\geq$ 9 with 10 M NaOH. After finishing MA addition, the reaction was allowed to proceed for 24 hours, after which the crude mixture underwent dialysis against distilled water for 2 hours (molecular weight cut-off 1 kDa, Carl Roth GmbH + Co. KG, Karlsruhe). Subsequently, water was replaced with dilute aqueous 0.01 M HCl at 4°C for one week, changing the acidic medium 1-2 times daily while protecting the dSIS-MA

from light. Post-dialysis, the dSIS-MA-containing solution was centrifuged at 1000 x g for 5 minutes to eliminate any residual particles if present. The supernatants were snap-frozen with liquid nitrogen and lyophilized (alpha 2-4 LDplus, Martin Christ Gefriertrocknungsanlagen GmbH, Osterode am Harz, Germany) to yield dry, cotton-like resins, which were stored at -20°C until use.

1.3. 3D printing of dSIS-MA-based Hydrogels

3D printing of dSIS-MA-based hydrogel discs was performed according to previously established protocols [4]. In brief, dry dECM-MA (1.5 wt%) was dissolved in 0.1 M NaOH and left to stand overnight at 4°C to achieve a homogeneous resin formulation for 3D printing. Immediately before printing, the photoinitiator LAP (1 wt%) was added to the resin formulation. To eliminate air bubbles from the mixing process, the resin was centrifuged at 140 x g for 5 minutes. The computer-aided design (CAD) models for the 3D discs (6 mm diameter, 1 mm height) were crafted using the 3D builder software (Microsoft Corporation, USA), then processed with the LongerWare software (version 1.35, LONGER, China) for slicing, and finally printed using a visible light liquid crystal display (LCD) printer (Orange 30, LONGER, China) at 405 nm with a light intensity of approximately 1 mW/cm^2 and a crosslinking duration of 50 seconds for each 100 µm layer. After printing, the 3D hydrogels were immersed in distilled water and washed 3 x 30 min to remove any unlinked macromolecules or photoinitiator. The hydrogels were then stored in PBS (-/-) at 4°C and used within two weeks.

1.4. Tissue Chamber Accessory

The tissue chamber was printed as described in the main article with the CAD files provided in the SI files. The transparent chamber lid made from a polycarbonate plate (thickness = 4 mm; Makroclear® from Arla Plast, Borensberg, Sweden) was fabricated by CNC milling with the dimensions shown below including four holes.

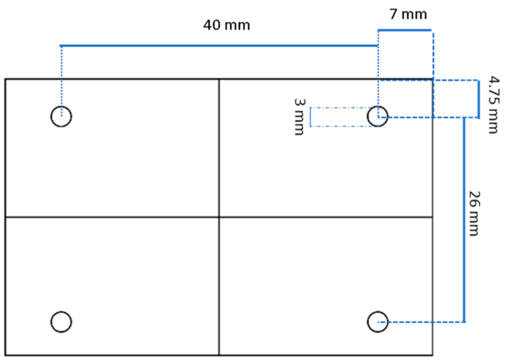

The medium reservoir is manufactured from a wide-mouthed, straight-walled polypropylene copolymer container (#10454631, PPCO, Nalgene™, Thermo Scientific™, USA) featuring a screw-on lid, available in various volumes to suit different experimental setups. For our experiments, we utilized the 15 mL container. These translucent containers offer superior chemical resistance and can withstand repeated autoclaving. Three 5.5 mm-diameter holes are drilled into the plastic lid using a drill (#2463977, Bosch Professional GSR 120-LI, Abstatt, Germany) and a drill bit (any brand). Threads (Threaded bit with 1/4" 28 UNF, any brand) are created to accommodate three adapters (for inlet, outlet, and gas exchange). The adapters (#40101, Threaded female luer lock connector; Qosina, Ronkonkoma, USA) are then installed and wrapped with poly(tetrafluoroethylene) (PTFE) tape to enhance sealing. A sterile 0.2 µm PTFE syringe filter (Minisart®, PTFE, 0.2 µm, #SRP15, Sartorius, Göttingen, Germany) is placed on the gas exchange adapter. The other two adapters are connected to tubing for integration into the circuit as described in the main article.

2. Material Characterization

2.1. dSIS-MA Resin Characterization

The presence of photoreactive groups, the degree of functionalization, and the protein's structural integrity of the dSIS-MA were determined via ^{1}H NMR spectroscopy, a chromogenic TNBS assay, and circular dichroism (CD) spectroscopy, in accordance with protocols reported previously [4].

2.2. Rheology of the dSIS-MA-based Hydrogels

Rheological analysis of the 3D-printed hydrogels was conducted following immersion in Milli-Q water for 24 hours at 4°C, with 2-3 water changes. Mechanical characterization was performed using a Kinexus pro+ oscillating rheometer (Malvern Panalytical) with an 8 mm parallel plate setup, a solvent trap, and a Peltier temperature controller (Anton Paar Physica, Ostfildern, Germany). The linear viscoelastic (LVE) regime was established through amplitude sweeps from 0.1 to 10% strain at 1 Hz, followed by frequency sweeps from 0.1 to 10 Hz at 0.5% strain at 37°C using samples pre-equilibrated in PBS (-/-) for 24 hours. Tests were conducted on three independent samples.

2.3. Atomic Force Microscopy (AFM) and Scanning Electron Microscopy (SEM) of dSIS-MA-based hydrogels

Morphology and surface stiffness of the 3D-printed hydrogels were characterized with a NanoWizard4® AFM (JPK Instruments, Berlin, Germany) equipped with a PetriDishHeater™ (JPK Instruments, Berlin, Germany) for *in situ* temperature-controlled measurements. The temperature was set to 37°C throughout the measurement.

To assess the morphology and surface Young's modulus E, a triangular silicon nitride pyramidal tip attached to a cantilever with a nominal spring constant (k) of 0.03 N/m and a tip radius of 20 nm (MLCT-D, Bruker, Mannheim, Germany) was used. Tip calibration and estimation of the spring constant were conducted by the thermal fluctuation method using the simple harmonic oscillator model on a bare petri dish and in PBS (-/-). For the nanoindentation experiments, a z-length of 3 μm, a speed of 2 μm/s, and a set point of 0.5 nN were used to ensure a very gentle indentation. Controlled deformations were applied to the samples and the compressive feedback forces were measured through cantilever deflection. Force-displacement (*F-z*) curves were produced by translating cantilever deflection (*d*) into force (*F*) by means of F= ¼ k*d*, where k is the cantilever spring constant. The Youngs modulus of the probed material was calculated by fitting the contact part of the measured approach force curves to a standard Sneddon model for a pyramidal indenter (tip) with a Poisson's ratio of 0.5. The measurements were operated in force spectroscopy contact mode, wherein an array 6 × 6 (36 points) of force-distance (*F–z*) curves was collected over the entire scan area of 40 x 40 μm. Morphological images of the samples were

obtained with the same cantilever in quantitative imaging mode (QI) using a set point of 0.5 V and a *z*-length between 1-2 µm scanning a 40 x 40 µm area of the hydrogel pixel-per-pixel.

SEM imaging was performed to visualize the inner and outer micro-architecture of 3D-printed hydrogel samples after lyophilization following our published procedure [4].

2.4. Human Crypt Isolation and Organoid Culture

Human intestinal crypts were harvested from the mucosa of the terminal ileum procured from endoscopic biopsies of healthy individual volunteers. The biopsies were repeatedly rinsed in cold PBS (-/-) until the supernatant cleared, then incubated in a chelating buffer (cf section 1.1) for 45 minutes on ice with gentle agitation. After removing the chelating buffer, crypts were liberated by vortexing the biopsies for 30 seconds in 10 ml of a modified chelating buffer without EDTA, DTT, and antibiotics. This vortexing step was repeated with fresh buffer three times to maximize crypt yield, with the resulting crypt-containing supernatants pooled and concentrated by centrifugation at 300 x g, 4°C for 5 minutes. The crypts were then washed thrice through centrifugation (400 x g, 4°C for 5 minutes) in basal medium (see section 1.1), resuspended in Matrigel™ diluted with basal medium (1:1 ratio), and plated in 50 µl droplets into a pre-warmed 24-well plate corresponding to ~100 crypts per well. The plates were first flipped upside-down, followed by a 20-minute Matrigel™ solidification at 37°C. Subsequently, the wells were supplemented with WRN-conditioned medium (expansion medium; see section 1.1) containing an initial supplement of 10 µM Y-27632 (RI) for the first two days. The medium was refreshed every 2-3 days with expansion medium without the RI supplement, and organoids were passaged weekly at a 1:6 ratio. For passaging, organoid-containing Matrigel™ domes were mechanically removed with a pipette tip from 12 wells of a 24-well plate, collectively placed into a 15 ml tube and supplemented with 6 ml cold basal medium (6 mL). Suspended organoids were then concentrated by centrifugation at 300 x g for 5 minutes at 4°C. After supernatant removal, the organoid pellet was enzymatically dissociated with 1.3 mL TrypLE™ supplemented with RI (10 µM) for 5 minutes at 37°C and subsequently subjected to mechanical disruption by expelling the solution through a blunt 18 G needle (10-20 times). The isolated cell pellet obtained after centrifugation (300 x g; 4°C; 5 min) was rinsed in cold basal medium (1 mL) and centrifuged once more (400 x g; 4°C; 5 min). After centrifugation and removal of the supernatant, the cells were resuspended in cold PCM medium (final density: 5x 10^5 cells/mL) and mixed with

Matrigel™ at a 1:1 ratio. The suspension was then placed as 50 µL domes per well into a pre-warmed 24-well plate with a flat bottom (2.5 x 10^5 cells/mL or 12.5 x 10^3 cells per Matrigel^{TM} dome), left for 3 min at 37°C before being flipped upside-down for 20-30 min 37°C to fully solidify the Matrigel$^{TM.}$ Next, 500 µL expansion medium is added per well with medium change every 2-3 days. Human terminal ileum-derived organoids of passage numbers 7 to 12 were used for the experiments.

2.5. Real-time Quantitative Polymerase Chain Reaction (RT-qPCR) Analysis

Sample preparation and RNA extraction: Cell culture medium was aspirated, and cellularized hydrogel scaffolds were gently rinsed with PBS (-/-) without disturbing the monolayer integrity. Subsequently, the cell-seeded scaffolds were placed into 1.5 ml Eppendorf tubes, lysed with TRIzol™ reagent (300 µl) and conserved at -80°C for later RNA extraction. RNA isolation was performed using Direct-zol RNA Microprep kit, according to the manufacturer's instructions. The isolated RNA was reconstituted in RNase-free water and maintained at -80°C. RNA purity and concentration were assessed using a NanoDrop™2000c spectrophotometer (Thermo Scientific™, USA).

Synthesis of cDNA from RNA and real-time quantitative PCR: To synthesize cDNA from RNA, 250 ng of RNA was first diluted in RNase-free water (10 µl). The solution underwent DNase I treatment (1 U) for 15 minutes at 22°C, which was subsequently inactivated with EDTA (25 mM) over 10 minutes at 65°C. The addition of Oligo(dT)$_{12\text{-}18}$ primers (12.5 ng/ml), dNTPs (10 mM), and supplemental water brought the volume to 40 µl, which was then incubated at 65°C for 5 minutes to allow primer annealing. Afterwards, RNaseOUT™ recombinant ribonuclease inhibitor (20 U) and DTT (10 mM) were introduced, followed by the addition of M-MLV reverse transcriptase (250 U) in first-strand buffer, with incubation periods of 50 minutes at 37°C and 15 minutes at 70°C, respectively. Post-reaction, samples were stored at 4°C.

For the qPCR analysis, 1 µl of the resulting cDNA was diluted with nuclease-free water, mixed with specific forward and reverse primers (**Table S2**), and SYBR™ Green PCR Master Mix according to the manufacturer's specifications. This reaction mixture was then placed into a MicroAmp™ fast optical 96-well reaction plate for processing in a StepOnePlus® real-time PCR system (Applied Biosystems, Thermo Fisher Scientific, USA), following these parameters: an initial denaturation at 95°C for 600 seconds, followed by 40 cycles of amplification (denaturation

at 95°C for 15 seconds, and annealing/extension at 62°C for 60 seconds each cycle). The specificity of amplification was confirmed through melting curve analysis. Relative gene expression was quantified using the expression of glyceraldehyde-3-phosphate dehydrogenase (GAPDH) as a reference gene, employing established comparative threshold cycle (Ct) methods.

2.6. DNA Content Quantification

To analyze the DNA content, 100 μL of the cell lysate was combined with PicoGreen™ working reagent from the Quant-iT™ PicoGreen™ dsDNA assay kit. Following a 5-minute incubation, the fluorescence was assessed using a microplate reader set to excitation/emission wavelengths of 480/520 nm. These fluorescence readings were then aligned against a standard dsDNA curve, facilitating the quantification of dsDNA content within each sample.

2.7. Immunofluorescence Staining

On predetermined days, epithelial cells from dynamic and static cultures were fixed after washing them twice with PBS (-/-) by incubation in 4% paraformaldehyde (PFA) at room temperature (RT) or cold methanol at 4°C for 10 minutes. Fixed cells were washed twice with PBS (-/-) and stored at 4°C until staining. For immunostaining, cells were permeabilized with 0.1% Triton X-100 for 10 minutes, blocked with 1% w/v BSA in 0.05% v/v Tween-20/PBS (-/-) for 1 hour and then incubated with primary antibodies for 1.5 hours. Afterward, secondary antibodies were applied for 30 minutes at RT in the dark. The specific primary and secondary antibodies utilized are listed in Table S1, including their dilutions in antibody buffer (1% w/v BSA in 0.05% v/v Tween-20/PBS (-/-)). Nuclei and actin filaments were stained with Hoechst 33342 (10 $\mu g \cdot mL^{-1}$) for 10 minutes and phalloidin-Atto®647 for 15 minutes in the dark, respectively. Following staining, samples were mounted and imaged with a confocal microscope (LSM800, Carl Zeiss, Jena, Germany) equipped with the Zen Blue software (v2.6, Carl Zeiss, Jena, Germany). 10x and 25x objectives (#420340-9901-000 EC Plan-Neofluar 10x/0.3 M27 and #420852-9871-000 LD LCI Plan-Apochromat 25x/0.8 Imm Corr DIC M27) were used for confocal imaging in one plane, while z-stacked 3D images were taken with the 10x objective with a slice thickness of 5-6 μm. Cells cultured on membranes were imaged on the membrane, while cells cultured on thick hydrogel samples (h = 1 mm) were flipped upside down for better image quality.

3. Supplementary Tables and Figures

Table S1. List of antibodies used in immunofluorescence staining.

Primary antibodies						
**Antibodies**	**ID**	**Company**	**Clonality**	**Host**	**Dilution**	**Fixation**
OLFM-4	AB85046	Abcam	Polyclonal	Rabbit	1:200	4% PFA
LGR5	AB273092	Abcam	Monoclonal	Mouse	1:200	4% PFA
Lysozyme	BS-0816R	Bioss	Polyclonal	Rabbit	1:200	4% PFA
Villin	PA5-29078	Invitrogen	Polyclonal	Rabbit	1:100	4% PFA
Chromogranin A	LK2H10	Invitrogen	Monoclonal	Mouse	1:200	4% PFA
MUC2	ABIN2854828	Antibodies	Polyclonal	Rabbit	1:200	4% PFA
MUC5AC	MA5-12178	Invitrogen	Monoclonal	Mouse	1:200	4% PFA
CD324	14-3249-82/DECMA-1	Invitrogen/e-bioscience	Monoclonal	Rabbit	1:100	4% PFA
KI-67	41-5698-80/SolA15	Invitrogen/e-bioscience	Monoclonal	Rabbit	1:200	4% PFA
ZO-1	40-2200	Invitrogen	Polyclonal	Rabbit	1:250	Cold Methanol
**Secondary antibodies**						
Goat anti-rabbit Alexa 647	A-21245	Invitrogen	Polyclonal	Rabbit	1:500	--
Goat anti-mouse Alexa 488	A-11001	Invitrogen	Polyclonal	Mouse	1:500	--

Table S2. Primers used in RT-qPCR analysis.

**Gene**		**Sequence 5' – 3'**
GAPDH	Forward	ACCCACTCCTCCACCTTTGAC
	Reverse	TGTTGCTGTAGCCAAATTCGTT
LGR5	Forward	GCGGGAAACGCTCTGACATA
	Reverse	CATCCAGACGCAGGGATTGA
ALPI	Forward	CATCCAGAAGGTCGCCAAGA
	Reverse	TGCCATTCTTCTGCCCCTTT
LYZ	Forward	GCCAAATGGGAGAGTGGTTA
	Reverse	TAACTGCTCCTGGGGTTTTG

MUC2	Forward	CAGAGGGCAGAACCCGAAAC
	Reverse	GAAGTTGTAGTCGCAGGGGC
CHGA	Forward	CTACGCGCCTTGTCTCCTAC
	Reverse	GCTGCCTGTGTTCAGAGAAG
MUC17	Forward	TGGGAGTGAGGAGATCCCAG
	Reverse	CGGGGCTTGTTTCCTGTACT
TFF3	Forward	TCCACCCGAGGACAGTTCTT
	Reverse	AGGGAGTCTTTTCCTGCCCT
MUC5B	Forward	GGCTGTTTCAGCACACACTG
	Reverse	CTTGTAGGTGGCCTCGTTGT
SI	Forward	TTTGGCAGCCTTATCCAAGTC
	Reverse	ACAGCAGGTGTCTTAGTTGCT
OLFM4	Forward	AAATGCTCGAGAGTTGCGGA
	Reverse	CACAGCAATCGTGTTGGTGG
MUC5AC	Forward	TATGTGCTGACCAAGCCCTG
	Reverse	TTGATCACCACCACCGTCTG

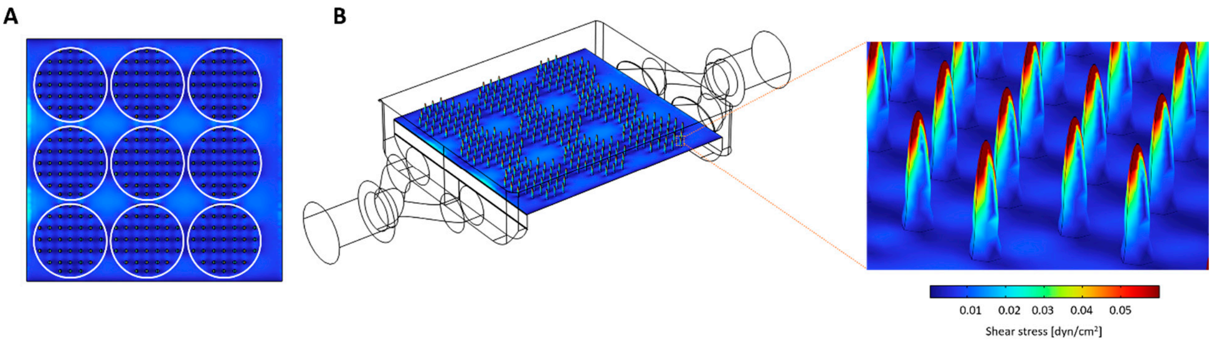

Figure S1. *CFD simulation of 3D villi-structured hydrogels inserted into the designed hydrogel-integrated millifluidic tissue chamber at a defined medium flow rate of 1.42 mL/min. The color gradient illustrates the shear stress distribution on the 3D structured hydrogel scaffolds made of 1.5% dSIS-MA. The modeled villi have a height of 1 mm and a diameter ranging from 0.28 mm at the base to 0.03 mm at the tip [2].* ***(A)*** *An xy projection of the hydrogel-integrated flow channel shows a consistent laminar shear stress distribution of approximately 0.01 dyne/cm² across the surface.* ***(B)*** *An xyz projection provides a closer look at the shear stress gradient along the villi, with shear stress values starting from ≤ 0.01 dyne/cm² at their base and increasing to ≥ 0.06 dyne/cm² at their tips.*

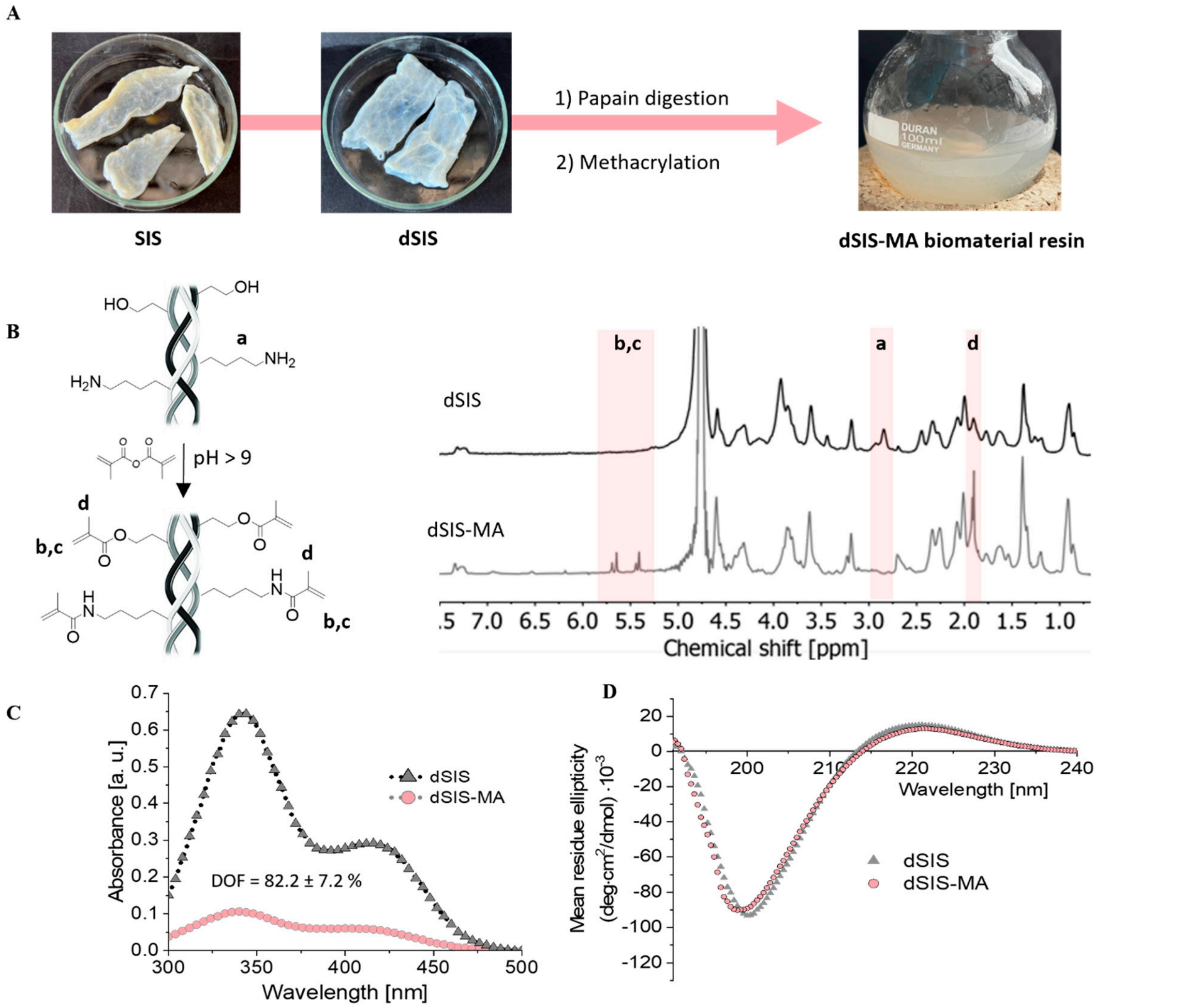

Figure S2. *Processing, modification, and characterization of dSIS-MA biomaterial resins for vat photopolymerization-based 3D printing of intestinal tissue hydrogels.* ***(A)*** *Photographic illustration of the processing of porcine SIS via decellularization to dSIS, enzymatic solubilization with papain, and subsequent chemical modification to obtain dSIS-MA resins.* ***(B)*** *Schematic illustration of methacrylation with methacrylic anhydride under aqueous basic conditions and comparative representative ^{1}H NMR spectra indicating the successful modification via the new signals attributed to the double bond (b,c) and the methyl group (d) as well as the vanishing methylene group signal (a) next to the free amino group.* ***(C)*** *Representative UV absorbance spectra of dSIS and dSIS-MA after reaction of their primary amino groups with the chromogenic reagent 2,4,6-trinitrobenzene sulfonic acid (TNBS) indicating a minimum degree of functionalization (DOF) with photocrosslinkable methacryloyl groups in dSIS-MA of around 82%*

*(n=3 of independent samples). **(D)** Representative CD spectra of digested dSIS and dSIS-MA indicating the preserved structural integrity of the proteins in dSIS-MA.*

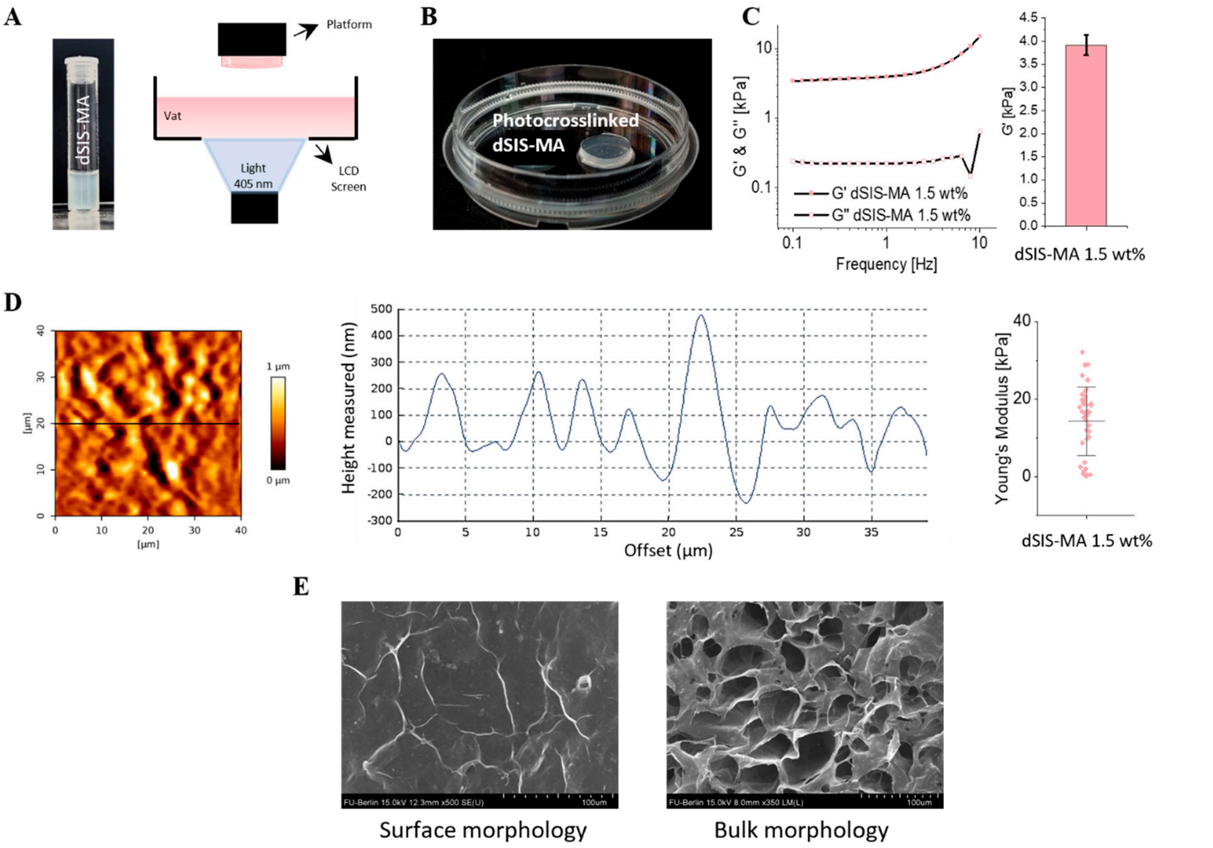

***Figure S3.** Characterization of printed dSIS-MA (1.5 wt%) hydrogels. **(A)** Photograph of the dSIS-MA (1.5 wt%) resin formulation and schematic illustration of the vat photopolymerization-based 3D printing process. **(B)** Photograph of the resulting flat and transparent printed hydrogel scaffold equilibrated in PBS (-/-) (d = 6 mm and h = 1 mm). **(C)** Representative storage and loss moduli G‘ and G“ of the printed hydrogel (dSIS-MA 1.5 wt%) as a function of frequency and derived stiffness of the gels at 1 Hz (n = 3, mean ± SD). **(D)** Surface topography, cross-section, and surface stiffness (Young's modulus) of the dSIS-MA (1.5 wt%) hydrogels accessed via AFM imaging in QI mode and nanoindentation, respectively (n=2, mean ± SD). **(E)** Surface and bulk morphology of the printed flat dSIS-MA (1.5 wt%) hydrogels via SEM imaging (n=2).*

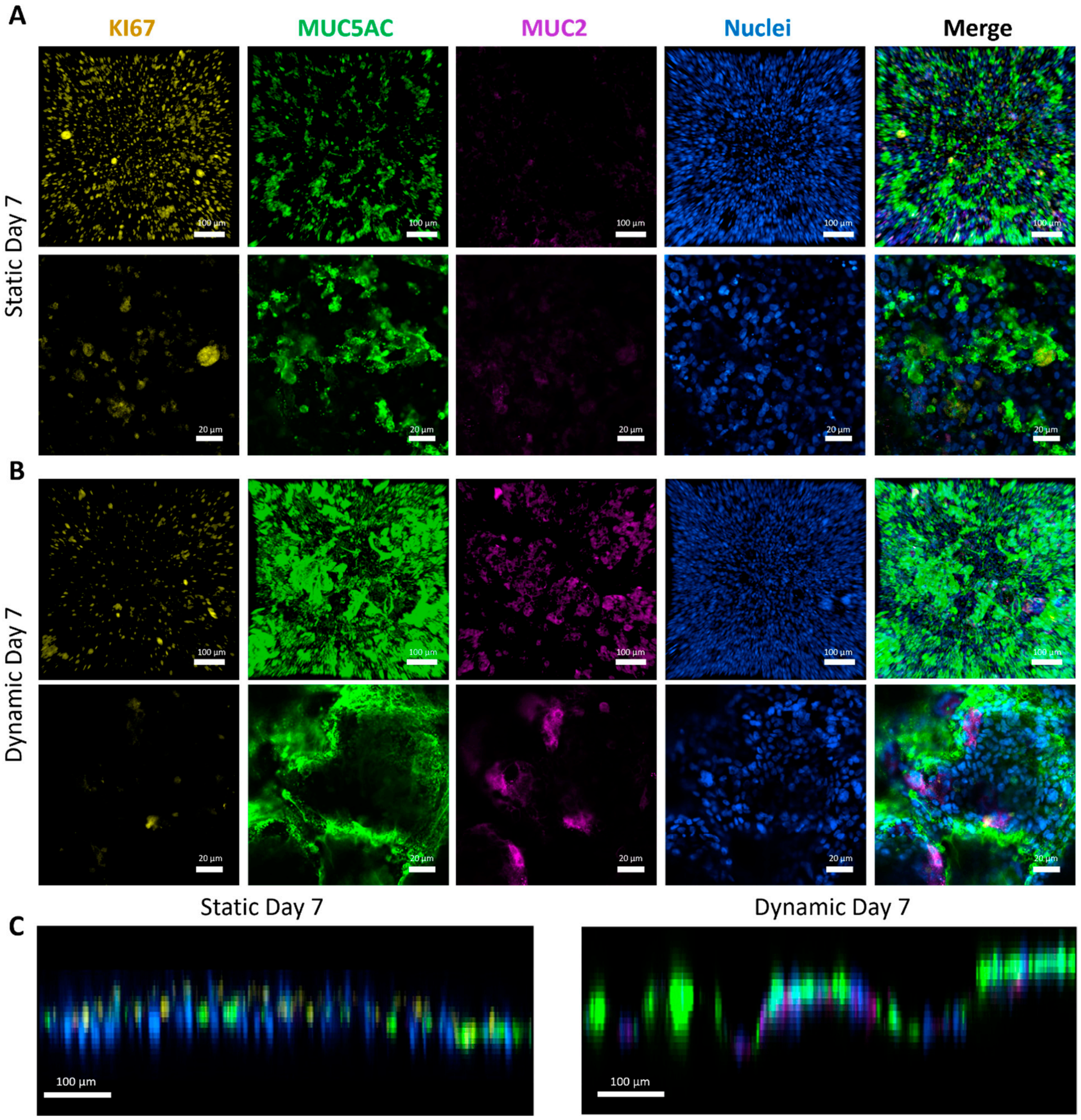

Figure S4. ***(A)*** *Representative confocal fluorescence microscopy images of HT29-MTX cells cultured statically on dSIS-MA (1.5 wt%) hydrogels for 7 days post-confluency. Immunofluorescence staining for KI-67 (yellow) indicates cell proliferation, MUC5AC (green) and MUC2 (purple) indicate mucins, and Hoechst 33342 staining indicates cell nuclei (blue). Top panel: xy projection of z-stack images; Bottom panel: higher magnification 2D images.* ***(B)*** *Representative confocal fluorescence microscopy images of HT29-MTX cells cultured under dynamic conditions (shear stress: 0.01 dyn/cm*^{2}*), stained and imaged with identical settings as*

*shown in **A**. **(C)** Side projections (xz/yz) of z-stacks from **A** and **B**. Representative images of three independent runs (n = 3) are shown.*

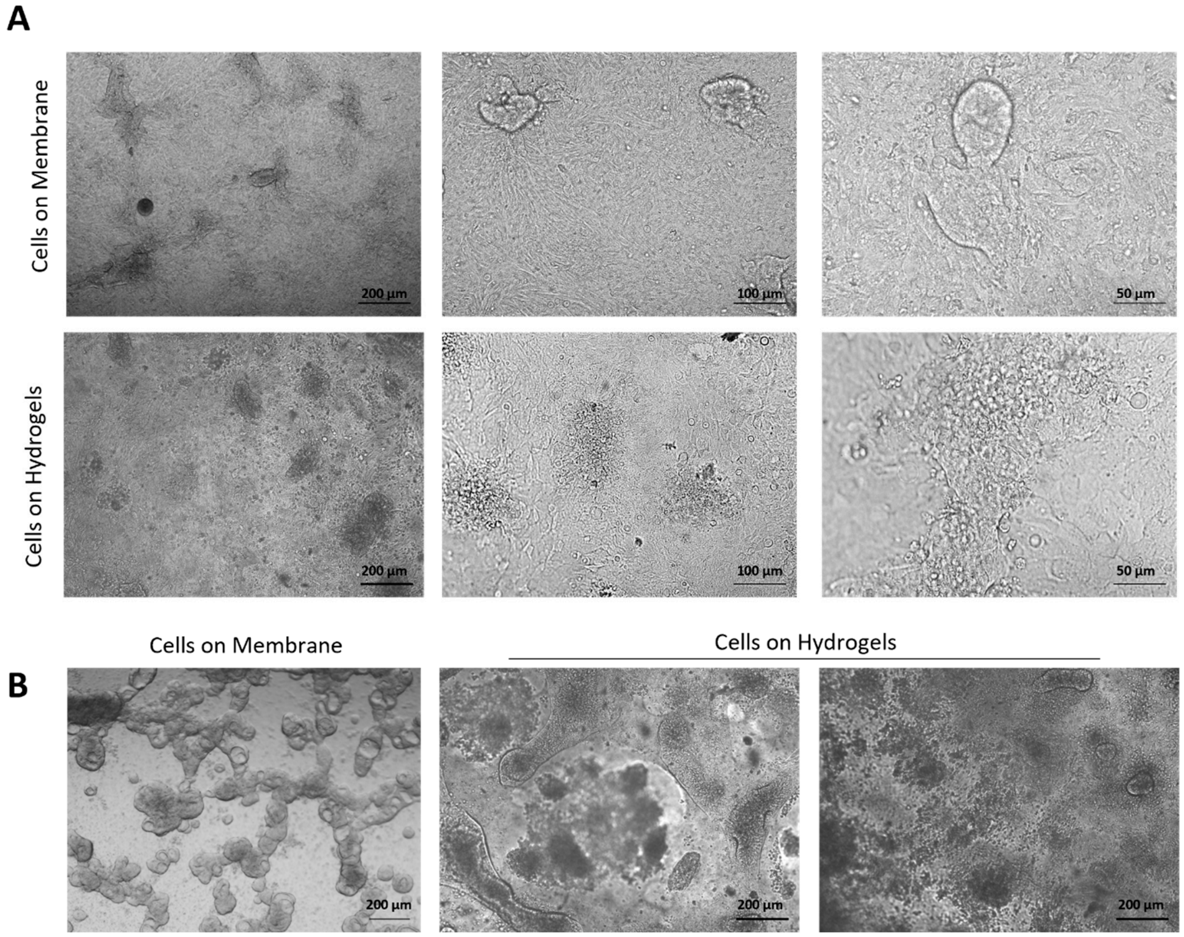

***Figure S5**. Representative phase contrast images of cell monolayers of human organoid-derived ISCs from the terminal ileum cultured statically in expansion medium on insert membranes coated with a thin film of Matrigel^{TM} and bulk uncoated dSIS-MA (1.5 wt%) hydrogel scaffolds. **(A)** Intact ISC monolayers imaged in three magnifications after reaching confluency (Day 0). **(B)** Exemplary images of culture trials on membrane and hydrogel, which did not yield fully confluent monolayers due to limited ISC proliferation even after extended culture periods, resulting in areas devoid of cells or cell debris. Such unsuccessful seedings in our hands comprise up to 40% of all trials.*

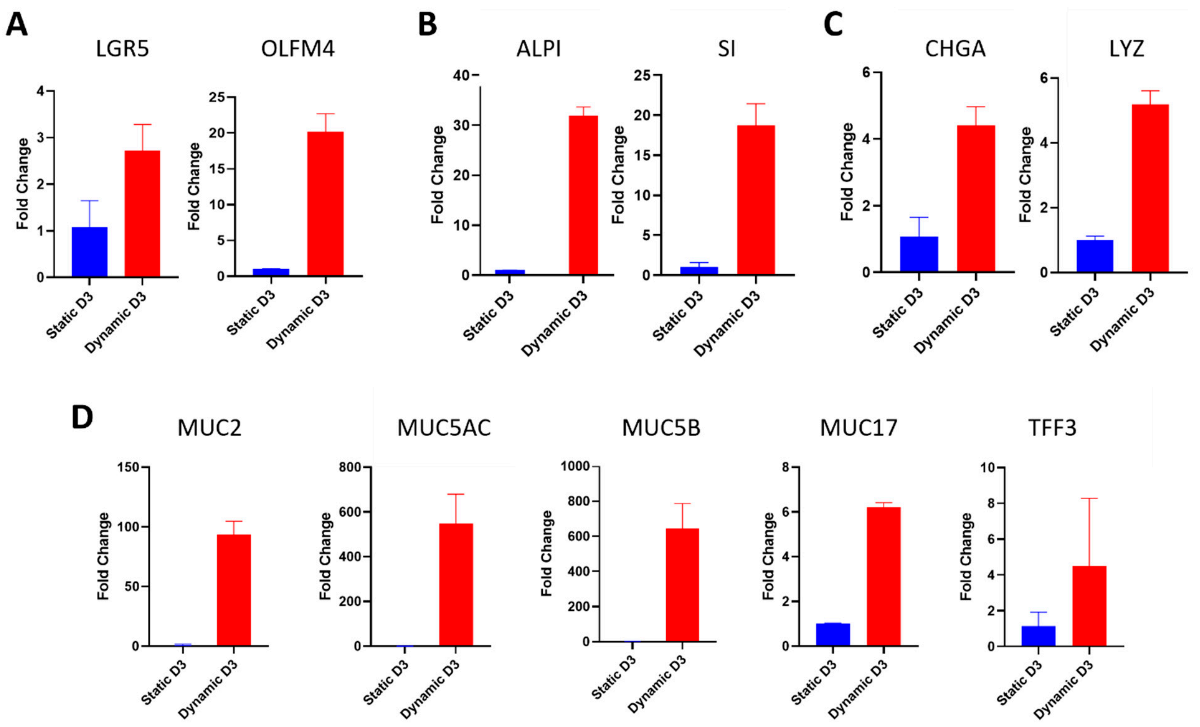

***Figure S6**. Quantitative RT-qPCR analysis of cells cultured on hydrogels in expansion medium under static and dynamic conditions assessed on Day 3 post-confluency showing the fold-change in transcript levels of **(A)** stem (LGR5) and progenitor (OLFM4) markers; **(B)** markers specific to enterocytes, namely alkaline phosphatase (ALPI) and sucrose-isomaltase (SI); **(C)** markers for enteroendocrine and Paneth cells, chromogranin A (CHRA) and lysozyme (LYZ), respectively; and **(D)** goblet cell markers MUC2, MUC5AC, MUC5B, MUC17, and TFF3. Data are presented as mean ± SD for n = 2 independent samples.*

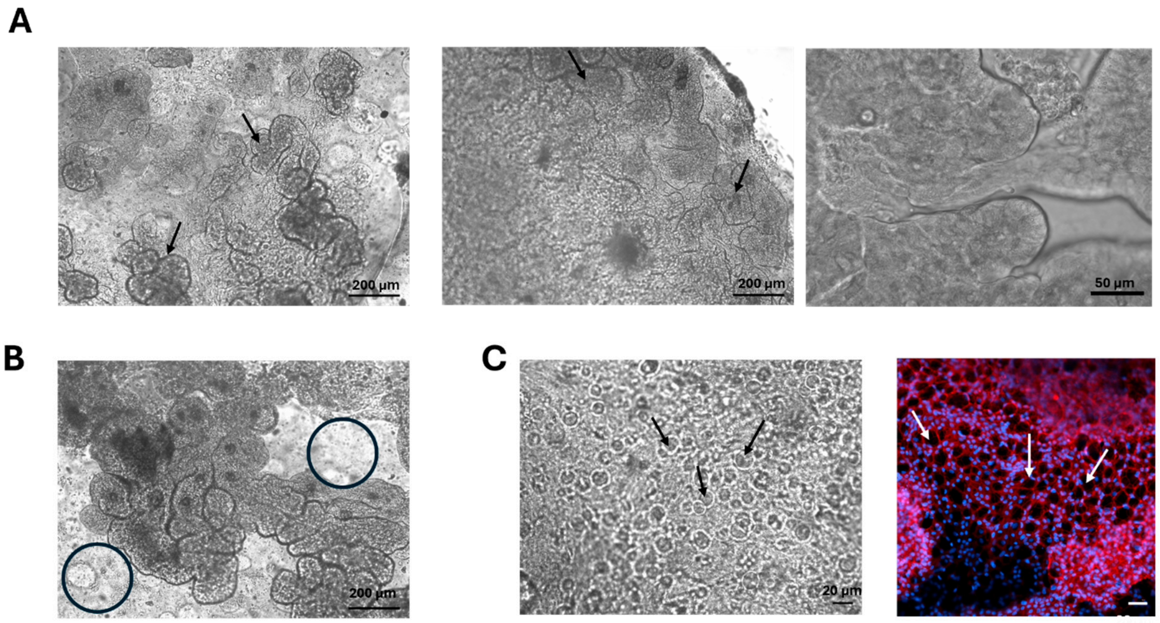

Figure S7. *Representative images of monolayers of human organoid-derived ISCs from the terminal ileum on dSIS-MA (1.5 wt%) hydrogels cultured in expansion medium and transferred to dynamic conditions for 5-7 days after reaching confluency. (A) Phase contrast images of intact cell layers with 3D villi-like protrusions (indicated by arrows), covering the entire surface of the hydrogel in different magnifications as predominantly observed. (B) Phase contrast images of cell layers with partial villi displacement, likely due to flow dynamics combined with diminished cell-hydrogel adhesion (indicated by circles) as rarely observed. (C) Rarely observed phase contrast and fluorescent images of comparably flat cell layers comprising a pore-like pattern ('pores' indicated by arrows) with representative fluorescent ZO-1 staining of such epithelial layers.*

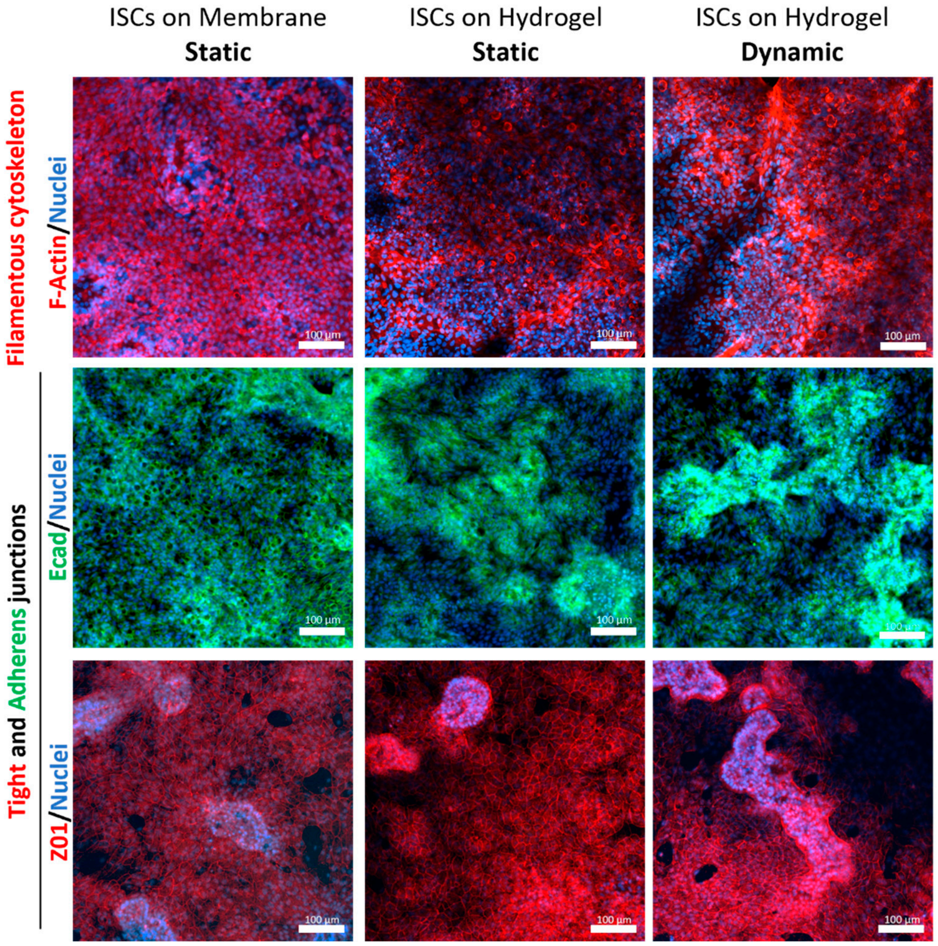

Figure S8. *Representative z-stacks of confocal fluorescence microscopy images of ISCs cultured on insert membranes and dSIS-MA hydrogels (1.5 wt%) statically or dynamically in expansion medium for 5-7 days after reaching confluency. IF staining of F-Actin (red), E-cadherin (green), and ZO-1 (red) indicates filamentous cytoskeleton as well as adherens and tight junctions with nuclei counterstaining (blue).*

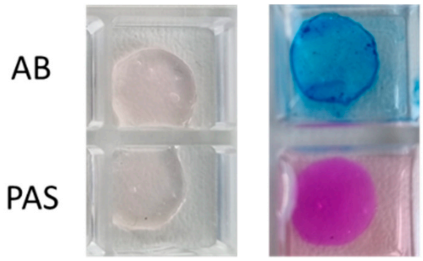

Figure S9. *Photographs of 3D-printed dSIS-MA (1.5 wt%) hydrogel scaffolds before (left side) and after (right side) incubation with alcian blue (AB) and periodic Schiff-base (PAS) reagents*

and subsequent water changes illustrating a significant AB and PAS background staining of dECM-based hydrogels.

4. Controller-Board Assembly and Pump Programming

4.1. Assembly of the Arduino Board to Control the Pump System

The following controller board-related pieces are purchased and assembled in the following order as illustrated in **Figure S11**:

A) First, the Arduino Uno microcontroller board based on the ATmega328P (Arduino Uno Rev 3, Arduino S.r.l, Monza, Italy) is assembled: The CNC shield V3 development board is attached (SIMAC Electronics GmbH, Neukirchen-Vluyn, Germany) for A4988 stepper drivers to control up to four stepper motors.
B) Next, the jumper pins (RM 2.54, BKL Electronic, Lüdenscheid, Germany) on the CNC shield are inserted to configure the X, Y, Z, and A axes for the pump stepper motors following Table S3.

Table S3. Jumper pins configuration of the X,Y,Z, and A axes for the step motors.

Axis	Definition (pins)
X	AccelStepper Xaxis(1, 2, 5)
Y	AccelStepper Yaxis(1, 3, 6)
Z	AccelStepper Zaxis(1, 4, 7)
A	AccelStepper Aaxis(1, 12, 13)

The motors are set to operate in quarter-step mode using the jumper pins attached to MS1-MS3 positions, balancing smoothness and torque, as outlined in the Table in **Figure S10** for standard micro-stepping.

A

Mode	MS1	MS2	MS3
Full step	Low	Low	Low
Half step	High	Low	Low
Quarter step	Low	High	Low
Eigth step	High	High	Low
Sixteenth step	High	High	High

B

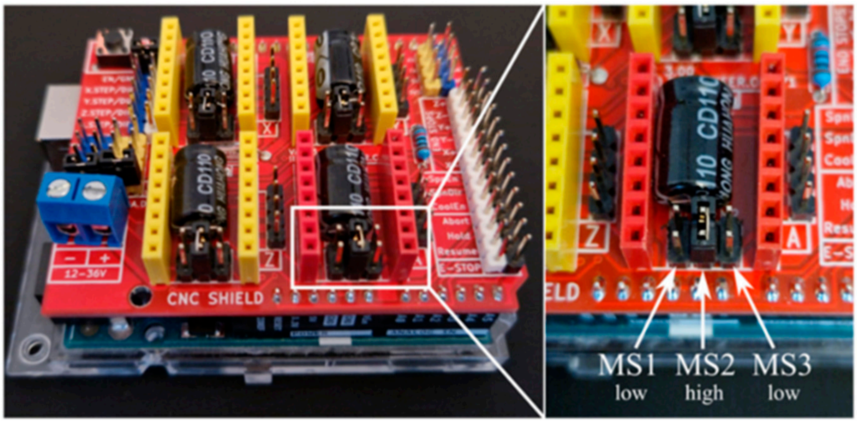

Figure S10. *(A) Table of the settings for different configuration modes of standard micro-stepping and (B) a photograph of the operational quarter step mode set on our board.*

C) Then, four carriers for A4988 stepper motor drivers are placed onto the CNC Shield (A4988, Pololu Robotics and Electronics, Las Vegas, NV/USA). 4-PIN Dupont cables (any brand) are used to connect the pump to the stepper motor drivers.

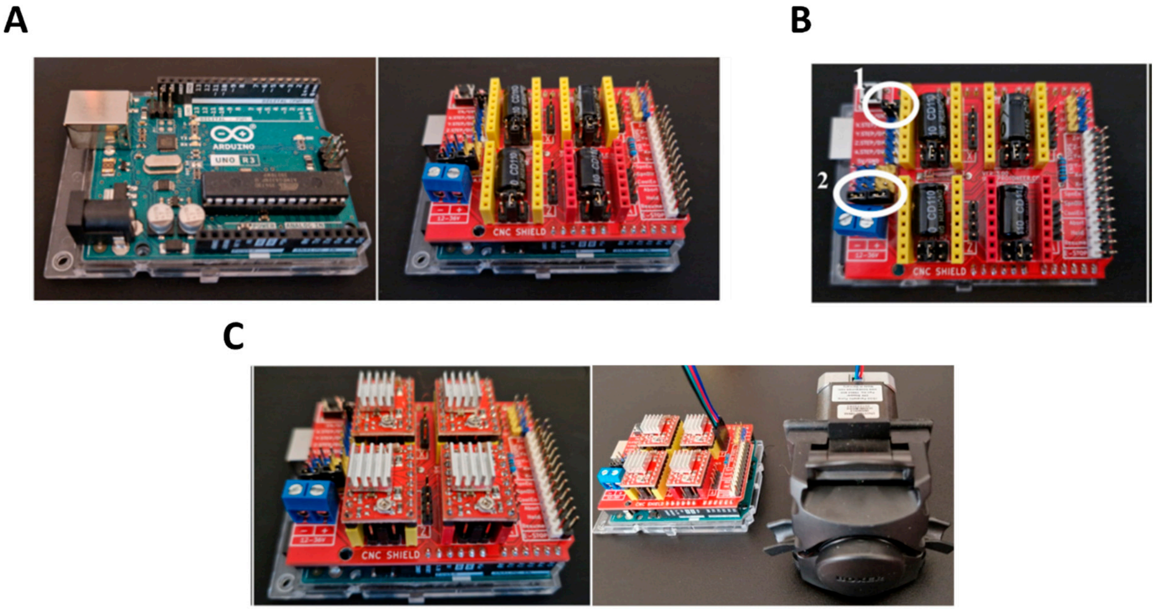

Figure S11. *Step-by-step assembly of the Arduino-controlled pump system*

The microcontroller can be powered via a normal USB cable (connected to a laptop or USB power adapter) or with a universal power supply (7-12 V, 1 A, plug: 5.5 x 2.1 mm).

Finally, before use, the reference voltage (V_{ref}) of the motor driver has to be adjusted based on the stepper motor's rated current and the stepper driver's sense resistor to prevent overheating or insufficient torque.

4.2. Arduino Software and Programming

A) Software installation and configuration

1. The free version of the Arduino IDE is installed from the official website (https://www.arduino.cc/) to write the script (sketch), which is intended to be operated by the microcontroller.
2. Within the IDE software, the AccelStepper library (https://www.arduino.cc/) has to be installed to manage the stepper motor control.
3. The provided sketch (see below) has to be loaded into the Arduino programming interface.
4. Finally, the revolutions per minute (RPM) settings must be fine-tuned for each axis to calibrate the desired flow rates.

B) Sketch (programming)

```
#include <AccelStepper.h>
  // 1  min = 60000
  // 5  min = 300000
  // 10 min = 600000
  // 15 min = 900000
  // 30 min = 1800000
  // 45 min = 2700000
  // 60 min = 3600000
float RPMXaxis = 12.; // speed of Pump connected to X-Axis
float maxStepsX = 1.; // number of steps for pump-speed increase; no
acceleration for a value of 1
unsigned long intervalLengthX = 1000; //in milliseconds, see above

float RPMYaxis = 12.; // speed of Pump connected to Y-Axis
float maxStepsY = 1.; // number of steps for pump-speed increase; no
acceleration for a value of 1; for example with 3 steps and 45 RPM: 1st
intervall runs with 15, 2nd with 30 and 3rd/final with 45 RPM.
unsigned long intervalLengthY = 10000; //in milliseconds, see above

float RPMZaxis = 12.; // speed of Pump connected to Z-Axis
float maxStepsZ = 1.; // number of steps for pump-speed increase; no
acceleration for a value of 1
unsigned long intervalLengthZ = 1000; //in milliseconds, see above

float RPMAaxis = 12.; // speed of Pump connected to A-Axis
float maxStepsA = 1.; // number of steps for pump-speed increase; no
acceleration for a value of 1; for example with 3 steps and 45 RPM: 1st
intervall runs with 15, 2nd with 30 and 3rd/final with 45 RPM.
unsigned long intervalLengthA = 10000; //in milliseconds, see above

//Do not change anything below this!!!

unsigned long delayStart = 0; // the time the delay started
// unsigned long currentStep = 0;

boolean doCalculationX = true;
boolean doCalculationY = true;
boolean doCalculationZ = true;
boolean doCalculationA = true;

//int displayX;
//int displayY;
//int displayZ;
//int displayA = 1;

// int led = 13; // not important for operation

AccelStepper Xaxis(1, 2, 5); // pin 2 = step, pin 5 = direction
AccelStepper Yaxis(1, 3, 6); // pin 3 = step, pin 6 = direction
AccelStepper Zaxis(1, 4, 7); // pin 2 = step, pin 5 = direction
AccelStepper Aaxis(1, 12, 13); // pin 3 = step, pin 6 = direction
```

```
void setup() {
//  pinMode(led, OUTPUT);   // initialize the digital pin as an output.
//  digitalWrite(led, HIGH); // turn led on

  delayStart = millis();   // start delay
  Xaxis.setMaxSpeed(1000.0);
  Yaxis.setMaxSpeed(1000.0);
  Zaxis.setMaxSpeed(1000.0);
  Aaxis.setMaxSpeed(1000.0);

//  Serial.begin(9600);
//  pinMode(10, INPUT_PULLUP);
//  pinMode(11, INPUT_PULLUP);

}
void loop() {

    if (doCalculationX) {
        unsigned long timeElapsed = millis() - delayStart;
        int intervalCountX = timeElapsed / intervalLengthX;
        if (intervalCountX < (maxStepsX - 1)) {
            Xaxis.setSpeed(RPMXaxis * 4 * (200. / 60.) * (intervalCountX + 1)
/ maxStepsX);
        } else {
            Xaxis.setSpeed(RPMXaxis * 4 * (200. / 60.));
            doCalculationX = false;
        }
    }
    if (doCalculationY) {
        unsigned long timeElapsed = millis() - delayStart;
        int intervalCountY = timeElapsed / intervalLengthY;

        if (intervalCountY < (maxStepsY - 1)) {
            Yaxis.setSpeed(RPMYaxis * 4 * (200. / 60.) * (intervalCountY + 1)
/ maxStepsY);
        } else {
            Yaxis.setSpeed(RPMYaxis * 4 * (200. / 60.));
            doCalculationY = false;
        }
    }
    if (doCalculationZ) {
        unsigned long timeElapsed = millis() - delayStart;
        int intervalCountZ = timeElapsed / intervalLengthZ;
        if (intervalCountZ < (maxStepsZ - 1)) {
            Zaxis.setSpeed(RPMZaxis * 4 * (200. / 60.) * (intervalCountZ + 1)
/ maxStepsZ);
        } else {
            Zaxis.setSpeed(RPMZaxis * 4 * (200. / 60.));
            doCalculationZ = false;
        }
    }
    if (doCalculationA) {
        unsigned long timeElapsed = millis() - delayStart;
```

```
        int intervalCountA = timeElapsed / intervalLengthA;

        if (intervalCountA < (maxStepsA - 1)) {
            Aaxis.setSpeed(RPMAaxis * 4 * (200. / 60.) * (intervalCountA + 1)
/ maxStepsA);
            //displayA = (RPMAaxis * 4 * (intervalCountA + 1) / maxStepsA);

        } else {
            Aaxis.setSpeed(RPMAaxis * 4 * (200. / 60.));
            doCalculationA = false;
        }
    }


    Xaxis.runSpeed();
    Yaxis.runSpeed();
    Zaxis.runSpeed();
    Aaxis.runSpeed();
}
```

References

1. Fairbanks, B.D.; Schwartz, M.P.; Bowman, C.N.; Anseth, K.S. Photoinitiated Polymerization of Peg-Diacrylate with Lithium Phenyl-2,4,6-Trimethylbenzoylphosphinate: Polymerization Rate and Cytocompatibility. *Biomaterials* **2009**, *30*, 6702-6707, doi:10.1016/j.biomaterials.2009.08.055.
2. Elomaa, L.; Gerbeth, L.; Almalla, A.; Fribiczer, N.; Daneshgar, A.; Tang, P.; Hillebrandt, K.H.; Seiffert, S.; Sauer, M.I.; Siegmund, B.; et al. Bioactive Photocrosslinkable Resin Solely Based on Refined Decellularized Small Intestine Submucosa for Vat Photopolymerization of in Vitro Tissue Mimics. *Addit Manuf* **2023**, *64*, 103439, doi:10.1016/j.addma.2023.103439.
3. Almalla, A.; Elomaa, L.; Bechtella, L.; Daneshgar, A.; Yavvari, P.; Mahfouz, Z.; Tang, P.; Koksch, B.; Sauer, I.; Pagel, K.; et al. Papain-Based Solubilization of Decellularized Extracellular Matrix for the Preparation of Bioactive, Thermosensitive Pregels. *Biomacromolecules* **2023**, *24*, 5620-5637, doi:10.1021/acs.biomac.3c00602.
4. Almalla, A.; Elomaa, L.; Fribiczer, N.; Landes, T.; Tang, P.; Mahfouz, Z.; Koksch, B.; Hillebrandt, K.H.; Sauer, I.M.; Heinemann, D.; et al. Chemistry Matters: A Side-by-Side Comparison of Two Chemically Distinct Methacryloylated Decm Bioresins for Vat Photopolymerization. *Biomaterials Advances* **2024**, *160*, doi:10.1016/j.bioadv.2024.213850.
